# Immunogenicity and protective efficacy of recombinant chimeric antigens based on surface proteins of *Toxoplasma gondii*

**DOI:** 10.3389/fimmu.2024.1480349

**Published:** 2024-12-13

**Authors:** Maciej Chyb, Bartłomiej Tomasz Ferra, Malwina Kawka, Marta Skwarecka, Bożena Dziadek, Justyna Gatkowska

**Affiliations:** ^1^ Department of Molecular Microbiology, Institute of Microbiology, Biotechnology and Immunology, Faculty of Biology and Environmental Protection, University of Lodz, Lodz, Poland; ^2^ The Bio-Med-Chem Doctoral School of the University of Lodz and Lodz Institutes of the Polish Academy of Sciences, Faculty of Biology and Environmental Protection, University of Lodz, Lodz, Poland; ^3^ Department of Tropical Parasitology, Institute of Maritime and Tropical Medicine in Gdynia, Medical University of Gdansk, Gdynia, Poland; ^4^ Pocket Diagnostics Poland, Gdansk, Poland

**Keywords:** recombinant chimeric antigen, *T. gondii* experimental vaccine, murine experimental model, *Toxoplasma gondii*, immunoprotection

## Abstract

**Introduction:**

Toxoplasmosis is caused by the opportunistic, cosmopolitan protozoan *Toxoplasma gondii* is one of the most common parasitoses in the world. This parasite can pose a threat to people with immunodeficiency but also to the fetus, since the invasion can lead to miscarriages. Moreover, this parasite can contribute to economic losses in livestock farming. These problems lead to the implementation of new, safe solutions for the development of effective toxoplasmosis immunoprophylaxis.

**Methods:**

In this work, newly produced recombinant trivalent chimeric proteins of *T. gondii*, based on SAG1-SAG2 recombinant chimeric antigen that differ in one terminal antigenic component, were tested in terms of their ability to induce an effective post-vaccination response. Antigens were tested *in vitro* to assess their ability to elicit APC cells response and further mice of the C3H/HeOuJ strain were immunized using those antigens, to evaluate their immunogenicity and immunoprotective effect *in vivo*. Two weeks after the last dose mice were either sacrificed to assess selected parameters of the immune response or infected with *T. gondii* DX strain to determine the degree of protection one month later.

**Results:**

The results of serological tests revealed a high level of serum IgG antibodies specific for the native *T. gondii* TLA antigens. TLA-stimulated splenocytes produced cytokines that are important in inhibiting protozoal invasion. Additionally, CD3^+^ CD4^+^ and CD3^+^ CD8^+^ T cell subpopulations of splenocytes were analysed by flow cytometry. One month after experimental infection mice were sacrificed, and their brains were isolated to count *T. gondii* tissue cyst. Immunization of mice with recombinant trivalent chimeric proteins of *T. gondii* resulted in reduction of tissue cyst burden rates reaching even 74%.

**Discussion:**

The obtained results demonstrate strong immunogenicity of the studied proteins and will allow to select candidates for further research aimed at increasing the immunoprotective properties of experimental vaccines against toxoplasmosis based on *T. gondii* chimeric antigens.

## 1 Introduction

Toxoplasmosis, one of the most common parasitoses worldwide caused by the invasion of the intracellular apicomplexan protozoan *Toxoplasma gondii*, which affects approximately 30% of the global population and is classified by the FAO/WHO as the fourth most important parasite and one of the most fatal foodborne pathogens in many countries (1). Despite being included in category III of zoonotic agents to monitor (1) it is also considered a neglected disease since it is often disregarded despite its impact on society and animal husbandry as the infection may prove fatal for immunocompromised individuals and for fetuses due to transplacental transmission (2, 3). The drugs routinely used in toxoplasmosis treatment are ineffective towards the latent parasite stage, which is responsible for infection reactivation and there are reports of increasing resistance of individual *T. gondii* strains to the treatments used for acute infection, and hence intensive research into new drugs continues (4, 5). Thus, vaccination would represent the most reliable and cost-efficient disease preventive measure. However, there is no specific immunoprophylaxis for human use and the only commercially available veterinary vaccine Toxovax, comprising live attenuated tachyzoites of S48 strain, has many limitations and can never be used in humans due to the risk of parasite reversion to fully virulent form (6, 7).

During primary infection in immunocompetent hosts the stimulation of immune system usually results in development of immunity capable of containing the acute invasion which leads to chronic phase. The protective immunity relies heavily on IFN-γ production which in turn is dependent on IL-12 released by activated macrophages, dendritic cells and neutrophils. Long-term protection involves activated CD4^+^ and CD8^+^ T cells which together with NK cells produce large quantities of IFN-γ to maintain the protective Th1 immune response and thus efficient vaccine should be able to induce timely production of IFN-γ and trigger strong protective immunity (8). Till today there have been many attempts at development of efficient anti-*T. gondii* vaccine, both for humans and veterinary purposes, involving different formulations, adjuvants, immunization routes and schedules, with the earliest employing live attenuated or killed parasites and crude fractions of *T. gondii* proteins e.g. whole cell lysate (TLA), excreted-secreted proteins (ESA). Modern approaches favor more standardized formulations based on recombinant proteins or nucleic acids (DNA, mRNA) (7, 9). Subunit vaccines represent the commonly used approach to immunoprophylaxis, comprising highly purified proteins and are considered very safe with a low chance of causing side effects in recipients. When administered with an appropriate adjuvant, they can induce protective immunity after immunization. The common consensus is that multi-antigenic formulations tend to be more efficient than vaccines based on single antigens, regardless of the vaccine type (10).

Thus, the aim of this study was to thoroughly evaluate the immunogenic and immunoprotective efficacies of four recombinant chimeric *T. gondii* antigens, chosen based on their antigenicity determined in previous studies (11). This approach to antigen selection for *in vivo* studies proved useful in our previous works. In the presented work we focused on improvement of protective capacity of tested antigens but also on implementation of complementary *in vitro* assays which could potentially prove helpful in the initial screening of possible antigenic compositions to choose the most promising ones for testing on experimental toxoplasmosis animal models.

## 2 Materials and methods

### 2.1 Animals

Animal experiments and procedures were performed in accordance with the ARRIVE 2.0 guidelines and approved by the Polish Local Ethics Committee for Experiments on Animals in Lodz (Agreements 8/ŁB159/2020 and 71/ŁD84/2017). They were also performed in accordance with the Polish Act on the Protection of Animals used for Educational and Scientific Purposes and in compliance with the European Directive 2010/63/EU of the European Parliament and of the Council of 22 September 2010 on the protection of animals used for scientific purposes.

Male C3H/HeOuJ mice (Charles River Laboratories) were bred in a specific pathogen-free (SPF) conditions in a facility at the Faculty of Biology and Environmental Protection, University of Lodz. Mice aged 8-12 weeks were used for the experiments, they were randomly assigned to the study groups, and kept 2-3 per cage under stable conditions: temperature 21°C +/-0.5, 55% humidity +/-5%, 12/12 h light/dark cycle, 15-20 air changes per hour, with free access to water and standard chow. The mice were transferred from the breeding rooms to the experimental laboratory 3-5 days before the start of the experiment to allow for acclimatisation. Mice were monitored daily during the experiment. Normal serum from Himalayan *Cavia prorcellus*, used as a source of complement, was obtained from animals bred in a facility at the Faculty of Biology and Environmental Protection, University of Lodz, under semi-barrier conventional conditions: temperature 20-22°C, 45-65% humidity, 12/12 h light/dark cycle, 15-20 air changes per hour, with free access to water supplemented with ascorbic acid and standard chow and hay. The serum was aliquoted and stored at -80°C until thawed only once for each experiment.

### 2.2 Parasites

Three strains of *T. gondii* were used to prepare the tachyzoite lysate: RH (ATCC-50174, ATCC, Manassas, VA, USA), RH-PRA (ATCC-PRA-310, ATCC, Manassas, VA, USA) and Me49 (ATCC-50611, ATCC, Manassas, VA, USA). *T. gondii* RH-GFP strain (ATCC-50940, ATCC, Manassas, VA, USA) was used to study macrophage phagocytic activity *in vitro*. The *T. gondii* DX type II strain was used for *in vivo* studies. RH-PRA, Me49, and RH-GFP strains were maintained *in vitro* on human foreskin fibroblasts Hs27 (ATCC- CRL-1634, Manassas, VA, USA) according to the manufacturer’s instructions. RH and DX strains were maintained *in vivo*. To obtain water-soluble whole-cell tachyzoite lysate antigens (TLA), the freeze-thaw technique was used as described previously (12, 13). Protein concentration was determined using commercially available Bradford reagent (Merck KGaA, Darmstadt, Germany).

### 2.3 Production of recombinant proteins

Recombinant plasmids encoding chimeric proteins were obtained as previously described (11). Recombinant chimeric proteins were produced using the *E. coli* strains Rosetta(DE3)pLysS. Production was carried out at 30°C for 16 hours after IPTG induction. Proteins were purified on a metal affinity Ni-Sepharose™ 6 Fast Flow column (Cytiva, Little Chalfont, England, United Kingdom) according to the manufacturer’s protocol in denaturing conditions. The purification resulted in electrophoretically homogeneous protein preparations, SAG1-SAG2 (aa: SAG1 49-311; SAG2 30-170; 50.15 kDa), SAG1-SAG2-MAG1 (aa: SAG1 49-311; SAG2 30-170; MAG1 30-222; 70.82 kDa), SAG1-SAG2-MIC1 (aa: SAG1 49-311; SAG2 30-170; MIC1 25-456; 96.3 kDa), SAG1-SAG2-P35 (aa: SAG1 49-311; SAG2 30-170; P35 26-170; 65.28 kDa), SAG1-SAG2-ROP1 (aa: SAG1 49-311; SAG2 30-170; ROP1 85-396; 83.69 kDa) with a purity of over 90%. Protein concentration was determined using commercially available Bradford reagent (Merck KGaA, Darmstadt, Germany). For *in vitro* studies, Pierce High Capacity Endotoxin Removal Spin Columns were used to remove potential LPS contamination using overnight manufacturer’s protocol, along with Pierce Chromogenic Endotoxin Quant Kit (Thermo Fisher Scientific, Waltham, MA, USA). Single recombinant antigens SAG1 (aa: 49-313, 33.42 kDa), SAG2 (AA: 30-170, 20.5 kDa), ROP1 (aa: 85-396, 39.11 kDa), MAG1 (aa: 30-222, 23.81 kDa), MIC1 (aa: 25-456, 52 kDa), P35 (aa: 26-170, 21.3 kDa) were obtained as previously described (14–18).

### 2.4 *In vitro* immunogenicity assay

For the *in vitro* assessment of antigen immunogenicity, two cell lines were used: human monocytes bearing the NF-κB-inducible SEAP reporter construct of THP1-Blue cell line (InvivoGen, San Diego, USA), and mouse macrophage cell line ANA1 (CVCL_0142, collection of the Department of Molecular Microbiology, Institute of Microbiology, Biotechnology and Immunology, Faculty of Biology and Environmental Protection, University of Lodz). NF-κB induction experiments were performed as described previously (19). Briefly, cells were cultured in RPMI 1640 medium (Gibco, Thermo Fisher Scientific, Waltham, MA, USA) supplemented with 2 mM L-glutamine, 25 mM HEPES, 10% heat-inactivated fetal bovine serum (Biowest, Cytogen, Zgierz, Poland), 100 μg/ml normocin (InvivoGen, San Diego, USA) and penicillin-streptomycin (100 U/ml - 100 μg/ml) (Merck KGaA, Darmstadt, Germany), and maintained at 37°C in a 5% CO_2_ atmosphere. Blasticidin (10 μg/ml, InvivoGen, San Diego, USA) was added as a repressor. Medium without blasticidin was used for assays. *E. coli* O55:B5 LPS (Merck KGaA, Darmstadt, Germany) was used as a positive control at concentration of 2.5 ng/ml. Cells alone (medium) were used as a negative control. After stimulation, supernatants were incubated with QUANTI-Blue reagent for 6 hours at 37°C. Optical density was read at 650 nm on SpectraMax i3 (Molecular Devices, San Jose, USA). Cell viability was assessed after each stimulation experiment using the resazurin reduction assay (**Supplementary Figure S2B**) to exclude false negatives due to potential antigen toxicity. To exclude false positive results due to contamination of antigen preparations by *E. coli* components, an *E. coli* Rosetta(DE3)pLysS culture was transformed with empty pET30 EK/LIC, induced and purified on a metal affinity column following the standard protocol for antigen purification. Metal affinity purified lysate was not purified on an endotoxin removal column. This sample was diluted as the least diluted recombinant antigen and used for cell stimulation (**Supplementary Figure S2A**).

ANA1 cells were maintained in DMEM medium (Gibco, Thermo Fisher Scientific, Waltham, MA, USA) suplemented with GlutaMAX (Gibco, Thermo Fisher Scientific, Waltham, MA, USA), 10% heat-inactivated fetal bovine serum (Biowest, Cytogen, Zgierz, Poland), penicillin-streptomycin (100 U/ml - 100 μg/ml) (Merck KGaA, Darmstadt, Germany), and plasmocin 5 μg/ml (InvivoGen, San Diego, USA), at 37°C in a 5% CO_2_. Medium without plasmocin was used for tests. Cells were seeded at a density of 1x10^5^ cells/well in a 96-well plate and cultured for 24 hours. ANA1 cells were then stimulated with recombinant antigens and TLA Me49 at a concentration of 10 µg/ml for 24 hours. *E. coli* O55:B5 LPS (Merck KGaA, Darmstadt, Germany) was used as a positive control at a concentration of 10 ng/ml. Cells alone (medium) were used as negative control. After 24h of culture, supernatants were collected and stored at -20°C. Cell viability was assessed after each stimulation experiment using the resazurin reduction assay (**Supplementary Figure S2B**) to exclude false negatives due to antigen toxicity. Simulated cells were infected using *T. gondii* RH-GFP strain 1x10^5^ cells/well. RH-GFP assay was performed in DMEM medium without phenol red (Gibco, Thermo Fisher Scientific, Waltham, MA, USA) supplemented with 2 mM L-glutamine, 3% double heat-inactivated FBS (Biowest, Cytogen, Zgierz, Poland), penicillin-streptomycin (100 U/ml - 100 μg/ml) (Merck KGaA, Darmstadt, Germany). 72 hours after infection, fluorescence reading was performed at 488/510 nm on SpectraMax i3 (Molecular Devices, San Jose, USA). The percentage of viable *T. gondii* was calculated, based on infected unstimulated cells values. Cytokine concentrations of TNF-α (Invitrogen, Thermo Fisher Scientific, Waltham, MA, USA), IL-12p40 (Invitrogen, Thermo Fisher Scientific, Waltham, MA, USA), and IL-10 (OptEIA, BD Biosciences, San Jose, CA, USA) were measured using commercially available ELISA kits according to the manufacturer’s instructions. Cytokine concentrations were calculated using non-linear regression.

Tests were performed using at least two different protein preparations of each antigen, each experiment included 5-8 technical replicates, which were averaged. NF-ĸB pathway activation and ANA1 cells cytokines production data are presented as mean of 3 or more independent experiments. *T. gondii* viability data are presented as a mean of 5 or more independent experiments.

### 2.5 Mouse immunization and challenge

Immunization and challenge experiments were performed as described previously (19–21).

Mice were first randomly assigned to cages, which were then randomly assigned to four groups: four mice per group for the splenocyte/serology assay and ten for the *T. gondii* challenge. They were then vaccinated subcutaneously three times at two-week intervals with recombinant chimeric antigens SS, SS-MAG1, SS-MIC1, SS-P35, and SS-ROP1 (10 μg/mouse) diluted in PBS and emulsified in incomplete Freud’s adjuvant (Merck KGaA, Darmstadt, Germany). Control animals received only PBS emulsified in adjuvant. Study groups were coded. Experiments on animals were performed by the same investigators, with only one investigator aware of the allocation of mice.

Two weeks after the last dose, mice (4 per group) were anaesthetised with sodium pentobarbital (intraperitoneal injection, 200 mg/kg) and euthanised by cervical dislocation for organ and tissue removal. Blood and spleens were used for serology and splenocyte assays.

The remaining 10 animals were challenged intraperitoneally with five *T. gondii* DX cysts per mouse. One month after *T. gondii* challenge, mice were sacrificed, the whole brain was isolated and mechanically homogenised in PBS supplemented with penicillin-streptomycin (100 U/ml - 100 μg/ml) (Merck KGaA, Darmstadt, Germany) and stored at 4°C. *T. gondii* cysts were counted at least in duplicate under an inverted light microscope and the mean cyst count was calculated for the volume of the whole homogenate. Cyst counts were performed by at least two experimenters, who were unaware of the experimental set-up, and counted samples randomly independent of the group.

### 2.6 Serology assays

ELISA assays were used to determine the levels of TLA RH specific IgG and IgM antibodies at 1:500 and 1:100 sample dilutions, respectively. The assay was performed as described previously (13, 19, 20) on serum samples collected from mice two weeks after the last dose of the vaccine. Briefly, 96-well MaxiSorp plates (Thermo Fisher Scientific, Waltham, MA, USA) were coated overnight (4°C) with TLA RH 2 µg/well in 0.1 M sodium carbonate buffer. Washes were performed with PBS/0.05% Tween 20. 10% heat-inactivated FBS/PBS was used for blocking and as an assay diluent. Each serum was tested in duplicate. The mean of duplicates of each sample was plotted. The reaction was developed with the secondary HRP-conjugated goat anti-mouse IgG or IgM antibodies (Jackson ImmunoResearch, West Grove, PA, USA), 2,2-azino-bis(3-ethyl-benzothiazoline6-sulfonic acid) diammonium salt 1 mg/ml (ABTS) (Merck KGaA, Darmstadt, Germany) and 0.0075% H_2_O_2_ serving as a chromogen and substrate, respectively, in 70 mM citrate-phosphate buffer pH 4.5. Using the same protocol, titrations were performed for IgG antibodies specific to the recombinant chimeric antigens used for the vaccination and single recombinant antigens constituting chimeric proteins. Minor modifications were made, such as using 0.25 µg/well of recombinant antigens and serum dilutions ranging from 1:1,600 to 1:1,638,400. IgG1 and IgG2a subclass titration was performed with chimeric antigens using the same protocol but with goat anti-mouse IgG1 (Bio-Rad, Hercules, CA, USA) and goat anti-mouse IgG2a (Bio-Rad, Hercules, CA, USA) antibodies and serum dilutions ranging from 1:100 to 1:1,638,400. The absorbance was measured at 405 nm (Multiskan EX, Thermo Fisher Scientific, Waltham, MA, USA). Titers were defined as the highest serum dilution with OD > 0.3.

The functional assay of the antibodies was performed using the *T. gondii* RH-GFP strain grown on the Hs27 cell line. The idea was to assess whether the antibodies were able to inhibit the parasite invasion of host cells *in vitro* and whether they affected classical pathway complement activation and associated cytotoxicity. Hs27 cells were seeded at a density of 1.5x10^4^ cells/well in DMEM medium (ATCC, Manassas, VA, USA), supplemented with 10% FBS (ATCC, Manassas, VA, USA) and penicillin-streptomycin (100 U/ml - 100 μg/ml) (Merck KGaA, Darmstadt, Germany). Cells were cultured for 72 hours to obtain a confluent monolayer for the test.

Sera from vaccinated and control mice were incubated at 56°C for 30 minutes to inactivate the complement. Sera were then diluted in test medium (DMEM without phenol red, with 3% double heat-inactivated FBS and penicillin-streptomycin 100 U/ml - 100 μg/ml) and sterile filtered with a 0.22 µm low-affinity protein syringe filters (Sartorius Stedim Poland Sp. z o.o., Poland). Diluted sera mixed with *T. gondii* RH GFP at a density of 2x10^6^ cells/ml, alone or with the addition of a stable external complement source (to ensure the same complement activity for all mouse serum samples), normal serum of *Cavia porcellus*. The final dilution of test sera in the mixture was 1:25 and complement was at 1:63. The dilution of sera and complement was chosen experimentally in preliminary studies and also by testing the effect of test sera and external complement on Hs27 cell lines by MTT assay (**Supplementary Figure S4A**). Complement alone had cytotoxic properties against *T. gondii*. Therefore, the test with *T. gondii* RH GFP was used to select a dilution that did not cause cytotoxicity alone (**Supplementary Figure S4B**).

*T. gondii* RH GFP was incubated with serum of test and control mice, with or without external complement, for one hour at 37°C, 5% CO_2_. *T. gondii* RH-GFP in medium alone, incubated as test samples, was used as a control.

Hs27 cells were then infected using samples prepared as described above. The final infection load was 4x10^5^ *T. gondii* tachyzoites/well. The culture was carried out for 72 hours and then the 488/510 nm fluorescence was read on SpectraMax i3 (Molecular devices, San Jose, USA). *T. gondii* 100% viability was based on cells infected with *T. gondii* tachyzoites that were pre-incubated in medium alone. Diluted serum samples in the medium enhanced the autofluorescence of the medium, therefore appropriate diluted serum background controls were included in the assay design. Each serum sample was assayed in triplicate and averaged.

### 2.7 Splenocyte assays

Splenocyte stimulation assay was performed as described previously (19, 21).

In brief, spleens were extracted from both immunized and control mice, and splenocytes were obtained through mechanical homogenization and erythrocyte lysis. Utilizing a TC20 automated cell counter (Bio-Rad, Hercules, CA, USA), the cell count, and viability were determined using the trypan blue exclusion method. Splenocytes were cultured in IMDM medium (Biowest, Cytogen, Zgierz, Poland) supplemented with 5% heat-inactivated FBS, along with 100 U/ml penicillin + 100 μg/ml streptomycin. These cultures (7.5x10^5^ cells/well) were stimulated in triplicate with *T. gondii* RH strain TLA antigen at a concentration of 10 μg/ml. Additionally, Concanavalin A was included as a positive control (**Supplementary Figure S6**/**Supplementary Table S1**) at a concentration of 2.5 μg/ml (Merck KGaA, Darmstadt, Germany), while the culture medium alone served as the negative control. Following an incubation period of 48 hours (for IL-2) or 72 hours (for IL-10 and IFN-γ) at 37°C with 10% CO_2_, supernatants were collected, and cytokine concentration was assessed using commercially available OptEIA ELISA sets (BD Biosciences, San Jose, CA, USA). Data were analysed using non-linear regression to calculate cytokine concentrations. Negative control cytokine value was subtracted from stimulated cells value, separately for each mouse. The values of three technical replicates for each mouse splenocyte cytokine response were averaged and plotted on a graph. After 96 hours of splenocyte stimulation, the MTT assay was performed to assess cell proliferation as previously (20).

Flow cytometry was used to assess CD3^+^, CD4^+,^ and CD8^+^ populations. Cells (1x10^6^) were centrifuged and resuspended in FC buffer (PBS/2% HI FBS, sodium azide 0.09%) and stained with antibodies against CD4 PE clone GK1.5 Rat, CD8a BV421 clone 53-6. 7 Rat, CD3e APC clone 145-2C11 Hamster (BD Biosciences, San Jose, CA, USA) or corresponding isotype controls suggested by the manufacturer: Rat IgG2a PE clone A95-1, Rat IgG2a BV421 clone R45-95, Hamster IgG clone A19-3. Antibodies were titrated from 1 µg - 0.1 µg per 1x10^6^ cells and the optimal concentration was used in experiments, 0.1 µg for CD4 PE and 0.3 µg for CD8 BV421 and CD3 APC. Staining was performed based on BD Biosciences Immunofluorescent Staining of Mouse and Rat Leukocytes protocol. Briefly, cells were stained in a U-bottom microtitre plate in 100 µl of FC buffer for 40 minutes, 4°C, in the dark. The cells were washed 3 times with 200 µl of cold FC buffer and transferred to FC tube in 500 µl of FC buffer for data acquisition.

FC measurements were done in the Flow cytometry lab of the Faculty of Biology and Environmental Protection, University of Lodz. Data acquisition was performed on 4 laser LSR II (BD Biosciences, San Jose, CA, USA) flow cytometer, which is calibrated daily, with 405 nm laser and 450/50 bandpass filter for BV421, 488 nm laser and 575/26 bandpass filter for PE, 633 nm laser and 660/20 bandpass filter for APC. A total of 10,000 singlet events were collected. Dublets were excluded based on FSC-A/FSC-H and SSC-A/SSC-H gates. FMO control was used to set up positive gates. CD3^+^ cells were gated using the histogram. CD4^+^ and CD8^+^ cells were gated based on CD3^+^/CD4^+^ and CD3^+^/CD8^+^ cross gate. Analysis was performed using FlowJo 10.9.0 software (BD Biosciences, San Jose, CA, USA).

### 2.8 Statistical analysis

Graphs and all statistical analyses were performed using GraphPad Prism 10.2.2 for Windows (Dotmatics, GraphPad Software, California, USA). The Shapiro-Wilk and D’Agostino-Pearson tests were used to assess the Gaussian distribution of the data and residuals, along with analysis of the Q-Q plots. The Brown-Forsythe test was used to test the equality of the group variances. Spearman`s test for heteroscedasticity was used in the case of two-way ANOVA analysis. All data are presented as mean and standard deviation (SD). Antibody titers were logarithmically transformed to attain normality. Alpha/Q was set at 0.05 for all statistical analyses. For data meeting the requirements of parametric tests, ANOVA analysis was performed. In the case of unequal variances, Brown-Forsythe and Welch ANOVA tests were performed instead. For data that did not qualify for ANOVA, the Kruskal-Wallis test on ranks was performed. Two-way ANOVA was used for the comparison of IgG1, IgG2a, IgG titers, and for the functional antibody assay. The *post hoc* test used for each data analysis is provided below the figures.

## 3 Results

### 3.1 *In vitro* immunogenicity assay

**Figure 1:**
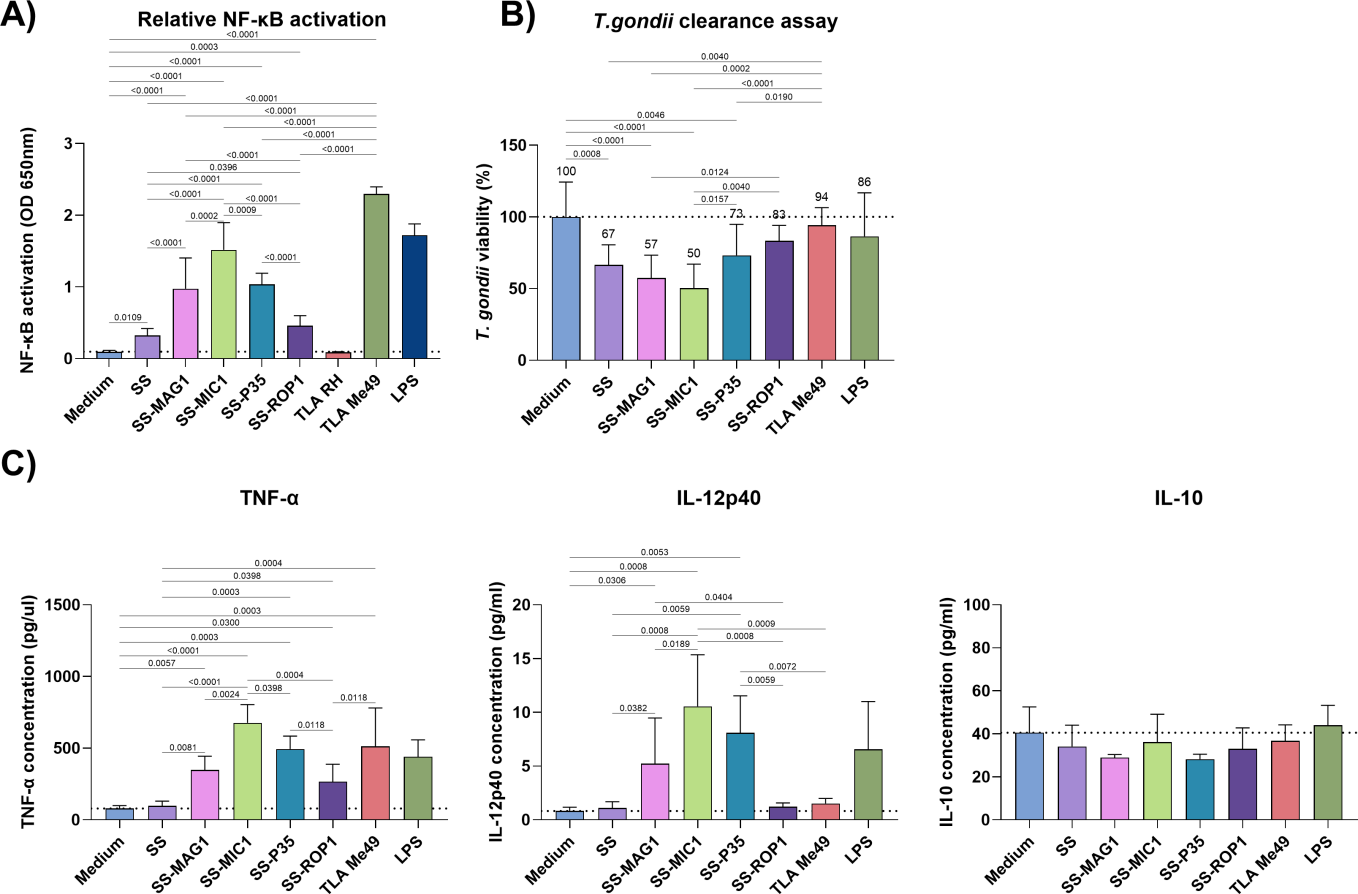
*In vitro* stimulation of APC cells. **(A)** Relative activation of the NF-κB pathway (OD 650 nm) after stimulation of THP1-Blue monocytes with recombinant chimeric antigens, TLA RH, TLA Me49, LPS or unstimulated cells (medium). Data are presented as mean and SD (n ≥ 3 independent experiments). **(B)** *T. gondii* clearance assay shows *T. gondii* RH GFP viability in recombinant chimeric antigen, TLA Me49, LPS (control) stimulated ANA1 macrophages or unstimulated cells (medium). Data are presented as mean and SD (n ≥ 5 independent experiments). **(C)** Selected cytokines production levels by ANA1 macrophages after stimulation with recombinant antigens, TLA Me49, LPS (control), or unstimulated cells (medium). Data are presented as mean and SD (n ≥ 3 independent experiments). The dotted line **(A-C)** represents the mean of the medium. Analysis for data **(A-C)** was performed using the one-way ANOVA followed by the FDR two-stage linear step-up procedure of Benjamini, Krieger, and Yekutieli to compare each mean with every other mean, excluding LPS. Q-value is shown above brackets.

The newly obtained recombinant *T. gondii* chimeric antigens, consisting of the bivalent chimeric antigen SAG1-SAG2 with the addition of a selected terminal antigen to form a trivalent chimeric antigen, were tested for their ability to induce a non-specific inflammatory response from APC cells, such as monocytes and macrophages. Two cell lines, human THP1-Blue monocytes, and mouse ANA1 macrophages, were used for these experiments. The monocytes used were carrying a reporter construct for activation of the pro-inflammatory NF-κB pathway. This allowed the relative level of activation of this pathway after contact with the chimeric proteins to be measured, compared to unstimulated cells (**Figure 1A**). Additional controls were a mixture of native *T. gondii* TLA antigens from RH and Me49 strains. To exclude potential false positives, purified *E. coli* lysate was also tested to reflect contamination of the preparation with *E. coli* components as shown in **Supplementary Figure S1A**. This showed no significant difference between baseline activation of the NF-κB pathway and that following contact with purified lysate. Each experiment was complemented by a determination of cell viability after stimulation, using a resazurin reduction assay, which confirmed that the antigens did not affect the viability of the cells used in the tests (**Supplementary Figure S1B**). All antigens tested activated the NF-κB pathway to a statistically significant degree compared to untreated cells (**Figure 1A**). An especially strong degree of activation was observed for the recombinant chimeric antigens SS-MAG1, SS-MIC1, and SS-P35. Of these, the SS-MIC1 recombinant chimeric antigen activated the pathway the strongest compared to all other antigens. The weakest activity was shown by cells stimulated with SS and SS-ROP1 recombinant chimeric antigens, whose activation levels differed slightly (SS-ROP1 > SS q-value = 0.0391) and were significantly lower than the other antigens. A mixture of native *T. gondii* antigens from the Me49 strain caused significantly high levels of activation of the NF-κB pathway (q-value < 0.0001), whereas TLA RH and TLA RH-PRA (results of both strains combined and labeled as TLA RH in **Figure 1A**) did not cause activation of this pathway, at the detectable level. The level of activation of the NF-κB pathway with TLA Me49 was significantly higher than that of all recombinant chimeric antigens tested.

ANA1 macrophages were also stimulated with the tested antigens. In this case, the focus was on the production profile of selected cytokines such as TNF-α, IL-12p40, and IL-10. As with human THP1-Blue monocytes, cell viability after stimulation was determined using the same method (**Supplementary Figure S1B**). The cytokine production profiles are shown in **Figure 1C**. The level of TNF-α production was significantly higher compared to unstimulated cells for the recombinant chimeric antigens SS-MAG1, SS-MIC1, SS-P35, and SS-ROP1. The level of production for these three antigens was significantly higher than for the SS and SS-ROP1 antigens, with an exception when comparing SS-MAG1 with SS-ROP1 (q-value = 0.1757). TLA Me49 stimulated TNF-α production by ANA1 cells, compared to unstimulated cells (q-value = 0.0003), SS (q-value = 0.0004), and SS-ROP1 (q-value = 0.0118) antigens. The SS-MAG1, SS-MIC1, and SS-P35 antigens induced significant IL-12p40 production by mouse macrophages compared to unstimulated cells. The level of production of this cytokine was highest for the SS-MIC1 recombinant chimeric antigen, significantly higher compared to SS-MAG1, SS, and SS-ROP1 of which the last two did not induce the IL-12p40 production. Similarly, TLA Me49 did not induce the production of this cytokine. Finally, none of the stimulants induced IL-10 cytokine production significantly above the level of unstimulated cells.

After stimulation with antigens, macrophages were infected with the *T. gondii* RH-GFP strain to determine the ability of the activated macrophages to eliminate the parasite. The results, expressed as a percentage of *T. gondii* viability, are shown in **Figure 1B**. Stimulation of macrophages prior to parasite contact with all recombinant chimeric antigens resulted in a decrease in *T. gondii* viability for each antigen, to 83-50%. A statistically significant decrease compared to unstimulated macrophages was observed for the SS, SS-MAG1, SS-MIC1, and SS-P35 recombinant chimeric antigens. Of these, *T. gondii* viability was lowest for the SS-MIC1 recombinant chimeric antigen, statistically significantly lower than for SS-P35 (q-value = 0.0157) and SS-ROP1 (q-value = 0.004) and averaging 50%. A similar result was achieved by the SS-MAG1 recombinant chimeric antigen with an average of 57%, significantly lower than SS-ROP1 (q-value = 0.0124).

### 3.2 Serology assays

**Figure 2:**
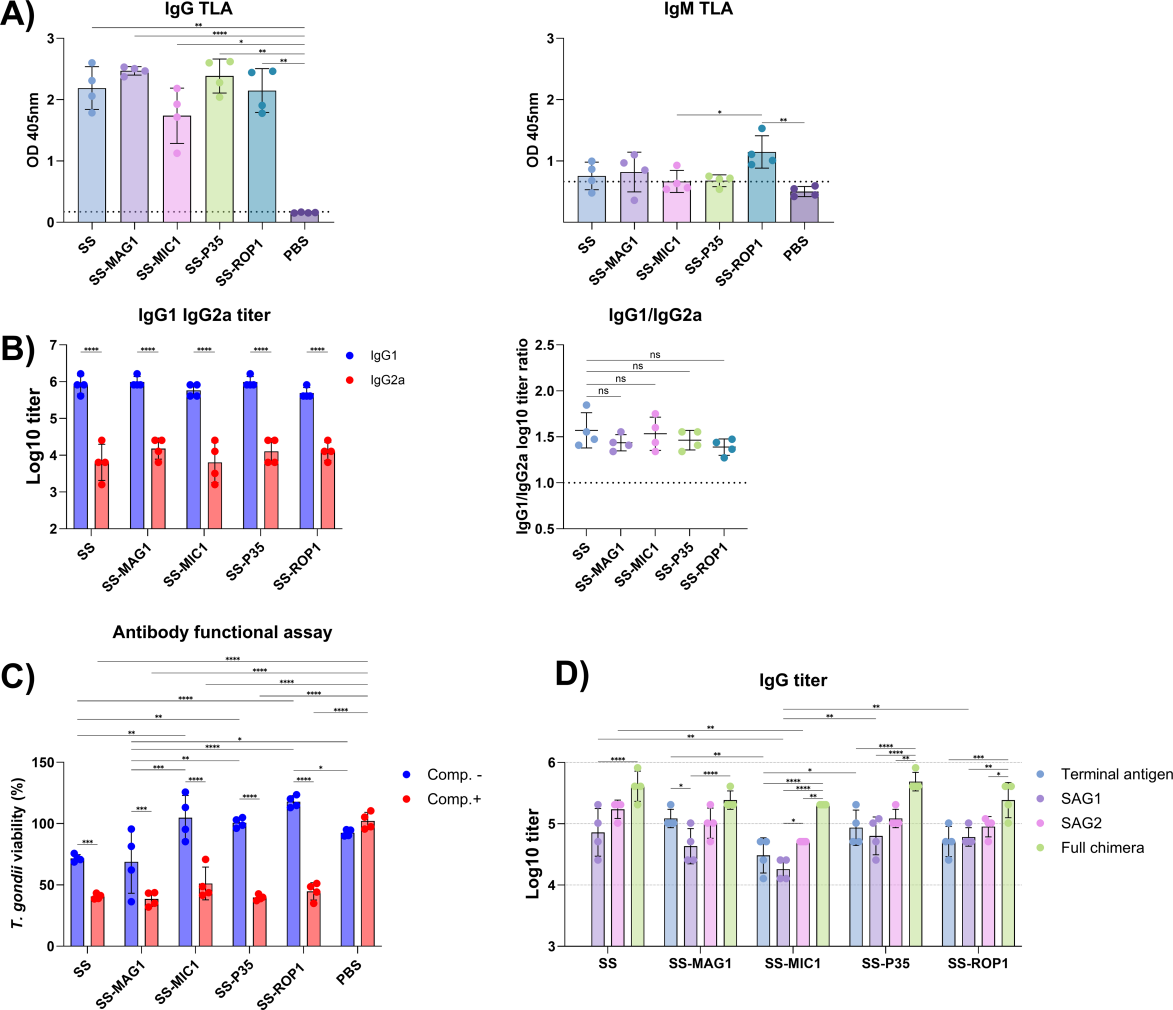
Antibody response following vaccination. **(A)** TLA RH specific IgG and IgM relative antibody levels. Sera dilution 1:500 **(IgG)** and 1:100 (IgM). The dotted line represents the cut-off value of the PBS group (mean + 2*SD). Data are presented as mean and SD (n = 4 mice). **(B)** Antigen specific IgG1 and IgG2a titers. Antibody titers were logarithmically transformed. Data are presented as mean and SD (n = 4 mice). IgG1/IgG2a graph shows the ratio of logarithmically transformed IgG1 and IgG2a titers. Data are presented as mean and SD (n = 4 mice). **(C)** Antibody functional assay indicates *T. gondii* viability after incubation with vaccinated and control (PBS) mouse sera in the presence (Comp.+) or absence (Comp.-) of complement factors. Data are presented as mean and SD (n = 4 mice). **(D)** Antigen specific IgG titer. Terminal antigen represents the single antigen used in a particular recombinant chimeric construct, such as MAG1 in SS-MAG1. Full chimera refers to the antigen used for vaccination. Antibody titers were logarithmically transformed. Data are presented as mean and SD (n = 4 mice). Analysis was performed using Brown-Forsythe and Welch’s ANOVA followed by Dunnett’s T3 multiple comparison test to compare each mean with every other mean of TLA RH specific IgG and IgM antibody level **(A)** and to compare each mean to the SS group of IgG1/IgG2a ratio **(B)**. Two-way ANOVA followed by Tukey`s multiple comparison test was used to compare IgG1 IgG2a titers within the antigen group and between the antigens within the antibody isotype **(B)**; to compare the means between each antigen with or without addition of complement factors, and to compare the means between Comp- and Comp+ in each antigen **(C)**; to compare the titers of individual chimeric component antigens within a group of test sera and between different groups of test sera within an individual chimeric component antigen **(D)**. ns – p_adj_ > 0.05, (*) – p_adj_ ≤ 0.05, (**) – p_adj_ ≤ 0.01, (***) – p_adj_ ≤ 0.001, (****) – p_adj_ ≤ 0.0001.

Mice of the C3H/HeOuJ strain were immunized with the tested recombinant chimeric antigens SS, SS-MAG1, SS-MIC1, SS-P35, and SS-ROP1. A series of experiments were carried out to determine the specific humoral response induced. The presence of IgG and IgM class antibodies recognizing native parasite antigens was determined. Relative antibody levels are shown in **Figure 2A**. Two weeks after the last dose, a significant level of IgG class antibodies recognizing the native TLA antigen was detected, with no statistical difference between the groups. In the case of IgM class antibodies, the SS-ROP1 group is characterized by the presence of IgM class antibodies in all animals tested, marked as dots on the graph. In the case of SS, and SS-MAG1 groups, it is evident that antibodies of this class are detected in 2-3 animals out of the group, above the cut-off of the test.

The titer of produced IgG class antibodies specific to the recombinant chimeric antigens used for vaccination as well as to the individual components of a given recombinant chimeric antigen was assessed (**Figure 2D**). Statistical analysis showed no significant differences in the titers of IgG antibodies recognizing the whole recombinant chimeric antigen between the different antigens. However, it is noticeable that the profile of IgG titers of the recombinant chimeric protein components is similar for each antigen. In each case, the mean titer of IgG antibodies recognizing the SAG1 antigen is lower than that of SAG2, while only SS-MIC1 showed statistical significance (p_adj_-value = 0.0244). For the SS-MIC1, SS-P35, and SS-ROP1 recombinant chimeric antigens, the titers of antibodies recognizing the whole recombinant chimeric antigen are significantly higher than the titers of the individual recombinant chimeric antigen components, whereas, for the SS and SS-MAG1 proteins, only the SAG1 titers differ significantly from those of the whole recombinant chimeric antigen (respectively p_adj_-value < 0.0001 and p_adj_-value = 0.0244). The lowest titers of antibodies recognizing SAG1 and SAG2 were found in the group immunized with the SS-MIC1 antigen, where SAG1 titers were significantly lower compared to SAG1 titers of the SS, SS-P35, and SS-ROP1 recombinant chimeric antigens, and antibody titers to the SAG2 antigen were significantly lower compared to the SS group. In general, the highest titers of IgG antibodies to SAG1 and SAG2 were found in the SS group. The lowest titers of antibodies to the terminal antigen were also seen in the SS-MIC1 group, with titers significantly lower than in the SS-MAG1 group. The titer of antibodies specific to the terminal antigen is highest in the SS-MAG1 group, in this one case the titer of IgG antibodies to the terminal antigen is significantly higher than the titer of antibodies to the SAG1 antigen.

One parameter to determine the polarity of the Th1/Th2 specific immune response is the determination of antigen-specific antibodies of the IgG1 (Th2) and IgG2a (Th1) isotype. For this purpose, IgG1 and IgG2a antibody titers specific to the antigen used for vaccination were determined (**Figure 2B**). High titers of IgG1 class antibodies, significantly higher than IgG2a class antibodies, were detected for each antigen. There were no statistically significant differences in the antibody titers of a given antibody subtype between the antigens tested. It was also confirmed that there were no significant differences between the IgG1/IgG2a ratios of antibody titers between antigens, although the highest ratio was observed for the SS recombinant chimeric antigen, reaching 1.571.

A functional antibody assay was performed to assess the ability of the post-vaccination specific antibodies to inhibit parasite invasion *in vitro*, either by themselves or through the ability to induce cytotoxicity via the classical complement pathway (**Figure 2C**). In this assay, specific antibodies (Comp.-) produced after immunization with SS and SS-MAG1 recombinant chimeric antigens were shown to significantly inhibit the parasite invasion *in vitro* by approximately 28% and 31%, respectively, compared to other antigens and, in the case of SS-MAG1, also to the PBS control group. When comparing the SS and PBS control groups, the adjusted p-value (0.0882) was close to the alpha level. The addition of an external source of complement to the sera of immunized animals resulted in a significant decrease in *T. gondii* viability compared to the PBS control group. For each antigen, the viability level of *T. gondii* decreased significantly after complement addition compared to the result without complement addition. After complement addition, the percentage of *T. gondii* inhibition was approximately 59, 62, 49, 60, and 55% for SS, SS-MAG1, SS-MIC1, SS-P35, and SS-ROP1 recombinant chimeric antigens, respectively. There were no statistically significant differences between groups after complement addition.

### 3.3 Splenocytes assays

**Figure 3:**
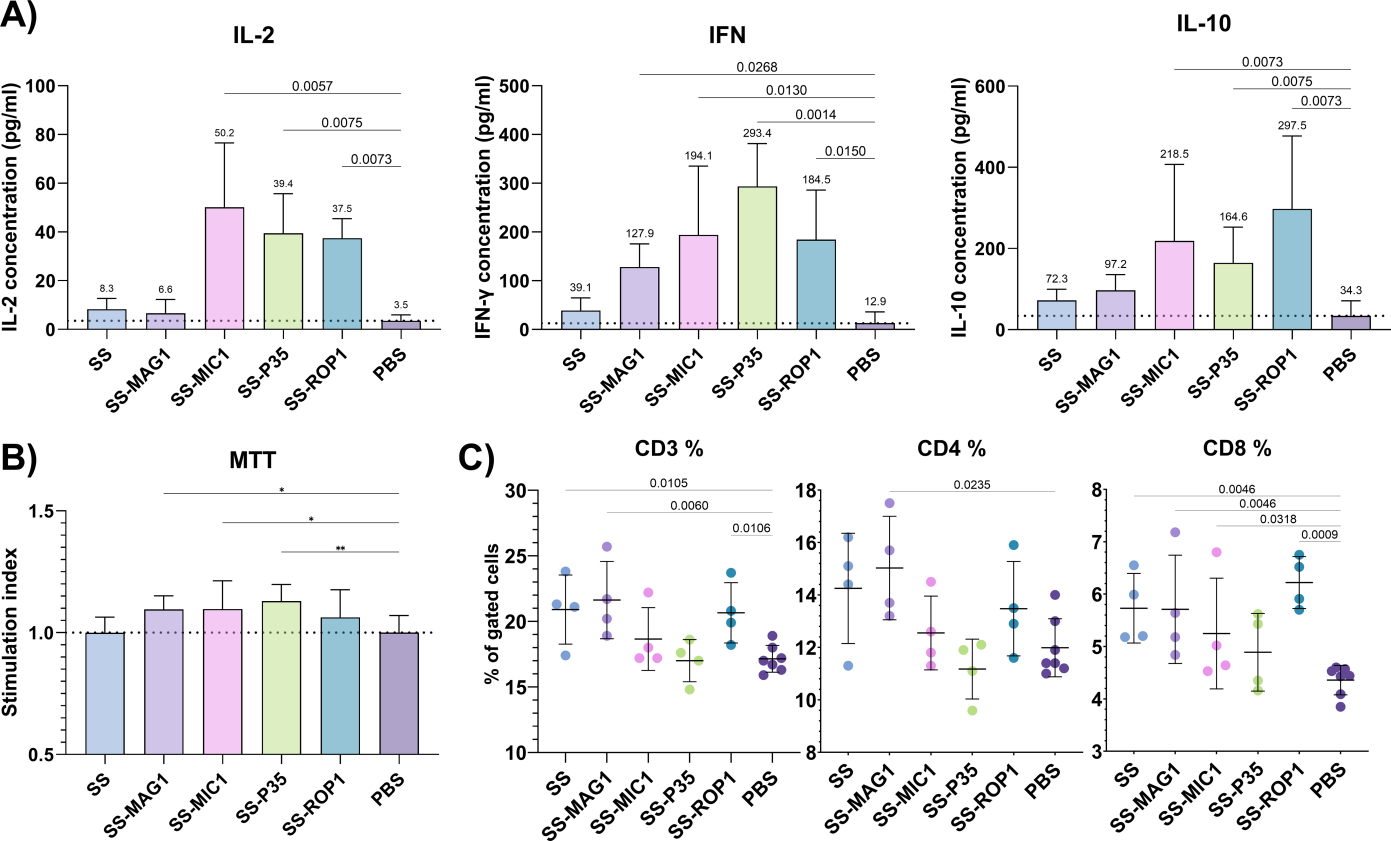
Post-vaccination cellular response. **(A)** Selected cytokine production levels by mouse splenocytes after vaccination. Data are presented as mean and SD (n = 4 mice). **(B)** Lymphoproliferation was assessed using the MTT assay and cell viability was calculated relative to unstimulated cells for each mouse, and then stimulation index (SI) was calculated based on antigen/PBS groups values. Data are presented as mean and SD (n = 4 mice). **(C)** Immunophenotyping of splenocytes after vaccination. Data are presented as mean and SD (n = 4-7 mice). The dotted line represents the mean of the PBS group **(A, B)**. Analysis was performed using one-way ANOVA followed by the FDR two-stage linear step-up procedure of Benjamini, Krieger, and Yekutieli to compare each mean with PBS as a control **(A, C)**. Q-value is shown above brackets. One-way ANOVA test followed by the Dunnet test to compare each mean of stimulation index with PBS as a control **(B)**. (*) – p_adj_ ≤ 0.05, (**) – p_adj_ ≤ 0.01.

To assess the cellular memory response after vaccination, a lymphoproliferation experiment was performed. Stimulation of splenocytes isolated from immunized mice with native *T. gondii* antigen TLA RH allowed to assess selected cytokine production levels (**Figure 3A**) as well as proliferation patterns (**Figure 3B**). Stimulation of splenocytes resulted in a significant upregulation of IFN-γ production in the case of SS-MAG1, SS-MIC1, SS-P35, and SS-ROP1 compared to PBS. Although there was a difference between the SS and PBS groups, FDR analysis did not show discovery. The same pattern was shown for IL-2 production except for the SS-MAG1 group where there were no statistically significant differences compared to the PBS group, in contrast to IFN-γ production. IL-10 production was also significantly higher in the case of SS-MIC1, SS-P35, and SS-ROP1 compared to the PBS group. Stimulation of splenocytes with TLA resulted in statistically significant splenocyte proliferation in the case of SS-MAG1, SS-MIC1, SS-P35, and SS-ROP1 recombinant chimeric antigens compared to the PBS control group.

Immunophenotyping was performed to assess the effect of vaccination on splenic T cell populations (**Figure 3C**). Vaccination with SS, SS-MAG1, and SS-ROP1 recombinant chimeric antigens significantly increased the percentage of CD3^+^ splenocyte populations. Vaccination with SS and SS-MAG1 recombinant chimeric antigens also significantly increased the percentage of CD3^+^CD4^+^ cells population. A statistically significantly higher percentage of the CD3^+^CD8^+^ cell population was shown for the SS, SS-MAG1, and SS-ROP1 groups. The difference between the antigen-induced profile of the CD3^+^, CD3^+^CD4^+^, and CD3^+^CD8^+^ populations was similar.

### 3.4 Cyst burden

**Figure 4:**
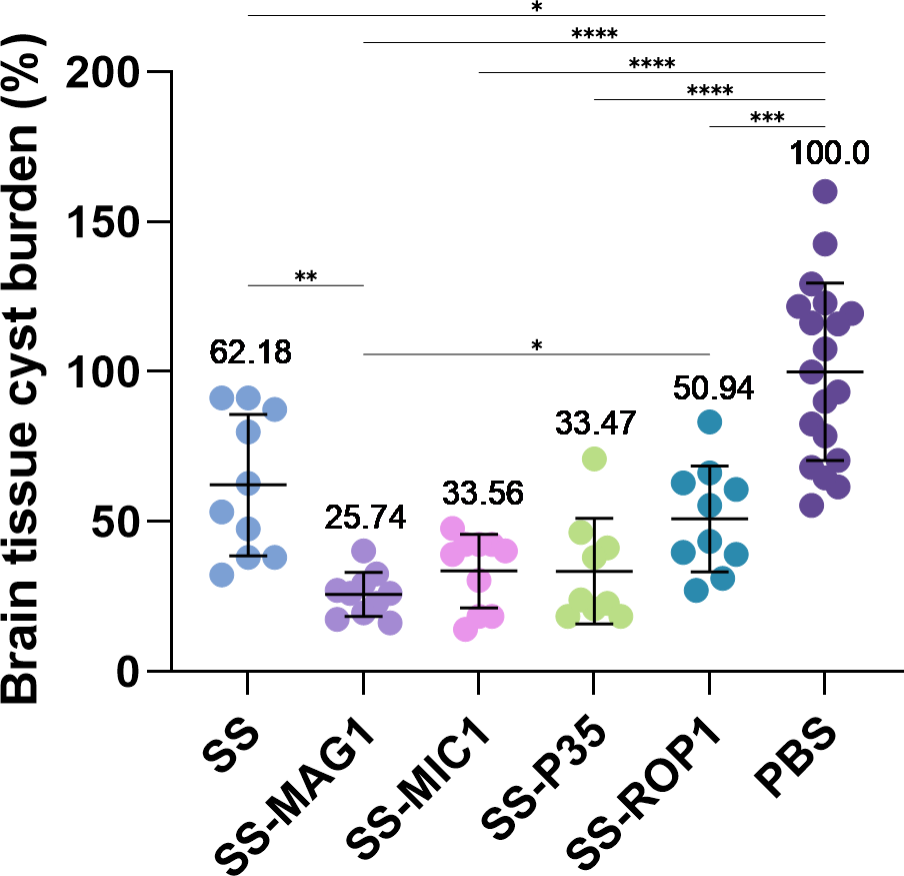
*T. gondii* tissue cyst burden in mice infected after vaccination. Data were calculated as the percentage of burden relative to PBS value. Data are presented as mean (value above dots) and SD (n = 10-20 mice). Analysis was performed using Brown-Forsythe and Welch’s ANOVA followed by Dunnett’s T3 multiple comparison test to compare each mean with every other mean. (*) – p_adj_ ≤ 0.05, (**) – p_adj_ ≤ 0.01, (***) – p_adj_ ≤ 0.001, (****) – p_adj_ ≤ 0.0001.

To evaluate the efficacy of the vaccine in inhibiting the development of chronic toxoplasmosis, animals were infected with the *T. gondii* DX strain. The reduction of parasite cysts tissue burden was determined as the degree of protection level achieved. Vaccination with each of the five recombinant antigens tested resulted in a statistically significant reduction in brain parasite load compared to the PBS control group (**Figure 4**, **Supplementary Figure S2**). The average reduction for SS, SS-MAG1, SS-MIC1, and SS-P35 was approximately 38, 74, 66, 67, and 49%, respectively. The highest percentage reduction for the SS-MAG1 group was statistically significantly higher than the SS and SS-ROP1 groups.

## 4 Discussion

*T. gondii* is one of the most common and widespread parasites in the human population. The threat posed by this parasite is often neglected because it is potentially harmless in immunocompetent individuals. Many research teams around the world have been working on the development of an effective anti-toxoplasmosis vaccine that could protect an individual from acquiring a latent form of toxoplasmosis that could cause problems later in life. The development of the vaccine itself is problematic in several ways, as described by other researchers (22).

Our research team has experience in testing various solutions for toxoplasmosis immunoprophylaxis (19–21). The current study was aimed at testing new chimeric recombinant *T. gondii* proteins composed of two *T. gondii* antigens fragments SAG1 and SAG2 (the SS core) and subsequently modified by the addition of a third terminal antigen as potential anti-*T. gondii* vaccines. From the seventeen SS recombinant chimeric antigens combinations thus developed, four were selected that exhibited a wide range of B cell epitopes, as demonstrated experimentally by their ability to efficiently bind specific anti-*T. gondii* antibodies in animal sera (11). These studies have shown that, among other things, these proteins have promising potential applications in the diagnosis of toxoplasmosis. All terminal proteins of 4 tested SS-based recombinant chimeric proteins play a key role in parasite’s life cycle (23–27). Single recombinant antigens MAG1, MIC1, P35, and ROP1 produced in *E. coli* were referred to as useful for serological diagnostics of toxoplasmosis (14, 17, 26, 28). Some of these, like MAG1 (29), MIC1 (30) or ROP1 (31) were used in vaccine trials with various efficiencies.

We began the assessment of the recombinant chimeric proteins immunogenicity by examining their ability to induce a non-specific inflammatory response, which can lead to the acquisition of a specific adaptive response.

Evaluating the immunogenicity of antigens by their ability to induce pro-inflammatory response of APC cells has previously been proposed as a potentially good screening tool for recombinant antigens (32–34). Our results showed that all the tested antigens induced NF-ĸB pathway in human monocytes (**Figure 1**), however, relative activation levels significantly differed between antigens. The relevance of this pathway in the induction of the adaptive immune response was discussed previously (35). For comparison, the lysate of two *T. gondii* strains, RH and Me49, was tested as a control of native antigens capacity to activate NF-ĸB pathway. The SAG1-SAG2 recombinant protein did activate the NF-ĸB pathway, although the level of activation is significantly lower compared to SS-MAG1, SS-MIC1, SS-P35, or SS-ROP1. SAG1 protein has already been reported as capable of inducing TNF-α secretion through the vimentin/PKCq-NF-kB pathway (36). It should be noted that chimeric antigens are not native proteins, therefore their immunogenicity may vary positively or negatively when compared to their native counterparts. In our case, the addition of MAG1 protein to the recombinant chimeric SAG1-SAG2 core increased the ability of this antigen to activate the NF-ĸB pathway. Interestingly, the MAG1 knock-out *T. gondii* strain did not change TNF-α production or NF-ĸB pathway activation compared to wild type when bone marrow-derived macrophages were infected (24). Not whole MAG1 antigen but only 30-222 amino acid residues were used to create SS-MAG1 antigen, while SS-MAG1 chimeric antigen containing 30-452 amino acids residues of MAG1 activated NF-ĸB pathway approximately 4 times weaker than the shorter version, and recombinant MAG1 (30-452 aa) antigen alone did not activate NF-ĸB pathway at all in human THP1 monocytes (**Supplementary Figure S5**). This suggests differences in MAG1 protein immunogenicity depending on the size and structure of the protein and potentially the availability of PAMPs.

Previous studies showed MIC1 ability to interact with N-glycans of TLR2 and TLR4, resulting in noncanonical carbohydrate recognition-dependent activation of innate immune cells and thus production of IL-12 (37). The team studying the detailed interactions of MIC1 and MIC4 proteins with host immune cells also confirmed the ability of MIC1 protein to activate the NF-ĸB pathway through TLR2 (38). In our case, the addition of MIC1 antigen (25-456 aa) to the chimera greatly enhanced the ability of a recombinant chimeric antigen to elicit NF-ĸB pathway activation.

Our results suggest that the addition of a surface antigen P35 (26-170 aa) or ROP1 (85-396 aa) antigen fragment to SS core, significantly increases the ability of the resulting protein to activate the NF-kB pathway. Interestingly, for the RH strain lysate TLA, no activation of the NF-ĸB pathway was detected, in contrast to the TLA Me49 which activated this pathway the most out of all stimulants used. However, it has been reported that type I strains like RH could inhibit activation of the NF-ĸB pathway, or activate it at low, undetectable levels, while type II strains like Me49 induced much stronger activation of this pathway (39). Again, we show that assessing NF-ĸB pathway activation can potentially be helpful in preselecting the antigen pool for further *in vivo* testing. It is therefore important to extend the proposed studies by examining antigens that are both more and less effective *in vivo* and *in vitro*, which would help to select predictive parameters for evaluating a given antigen’s effectiveness.

In this research, we extended the panel of *in vitro* immunogenicity tests with experiments on mouse macrophages of the ANA1 line. In this case, the production of cytokines such as TNF- α, IL-12p40, and IL-10 (**Figure 1C**) was tested. In addition, we were interested in whether antigen-primed macrophages had enhanced phagocytic activity and augmented *T. gondii* clearance (**Figure 1B**). ANA1 macrophages did produce high amounts of TNF-α and IL-12p40 in response to SS-MAG1, SS-MIC1, and SS-P35 stimulation. SS antigen did induce very faint, not significant TNF-α production compared to non-stimulated cells. TNF-α production profile of mouse macrophages looks similar to NF-ĸB pathway activation in human monocytes, however we notice here that TLA Me49 yields weaker effect, than in case of NF-ĸB pathway activation, compared to recombinant antigens. IL-12p40 production profile by mouse macrophages also resembles NF-ĸB pathway activation in human monocytes with differences that SS, SS-ROP1 and TLA Me49 antigens did not induce IL-12p40 production at all. The results of TLA Me49 stimulated macrophages are interesting since we saw high NF-ĸB pathway activation in TLA Me49 treated human monocytes. Previous studies showed that active *T. gondii* RH infection of mouse bone marrow derived macrophages causes IL-10-independent STAT3 activation, resulting in suppression of TNF-α and IL-12p40 production (40). However, the phenomenon was not shown for heat-killed parasite or soluble parasite lysate. Differences between studies might be due to macrophage line used or more likely by the differences between *T. gondii* strains. Later it was found that ROP16 kinase causes STAT3 activation (41). SS-MAG1 and SS-P35 induce significant levels of IL-12p40 while the SS-MIC1 chimeric antigen, induced the highest level of IL-12p40 production. Other authors also reported activation of NF-ĸB pathway but not IL-12 production in the case of some antigens (33). None of the recombinant antigens stimulated IL-10 production, suggesting an M1 phenotype, evident with SS-MAG1, SS-MIC1, and SS-P35 and less convincing in the case of SS and SS-ROP1. After stimulation with tested antigens, macrophages were infected with the *T. gondii* RH-GFP strain to study the effect of stimulation on their ability to phagocytise and combat the parasite, since it is known that this parasite has mechanisms to evade the microbicidal activity of macrophages, with non-activated macrophages becoming carriers of *T. gondii* in early stages of infection (42). The SS, SS-MAG1, SS-MIC1, and SS-P35 stimulated macrophages significantly reduced the number of tachyzoites compared to non-stimulated macrophages, resulting in an average of 33%, 43%, 50%, and 27% reduction in *T. gondii* viability, respectively. SS-ROP1 antigen-stimulated macrophages caused a non-significant *T. gondii* viability decrease, while it was still higher than in the case of TLA Me49, which may be related to the whole parasite native antigen mixture inhibiting phagocytic activity. Our findings are particularly important because of the growing interest in the development of memory macrophages via the IFN-γ pathway. Innate immune memory macrophages are more responsible for modifying inflammatory responses than phagocytosis, nevertheless, M1-like trained memory macrophages are of great importance for host defence and activation of innate immune cells by inflammatory mediators upon secondary infections (43, 44).

Mice immunized with the tested proteins produced significantly high titers of IgG antibodies recognizing the antigens used for immunization (**Figure 2D**). More importantly, these antibodies recognized native parasite antigens (**Figure 2A**). Two weeks after the last vaccine dose, we only detected IgM antibodies recognizing native antigens contained in TLA in single individuals, while the average result for the entire group was close to the cutoff value of the test except for the SS-ROP1 group, in which all four individuals still had specific IgM antibodies present in their sera. In the case of IgG1 and IgG2a subclasses, each antigen mainly induced the production of the IgG1 isotype associated with the Th2 response (**Figure 2B**). There were no significant differences in the IgG1/IgG2a titer index between the SS antigen and its modifications. Comparing these results with our previous studies, we note here a much stronger Th2-type response, comparing this with, other antigens administered with incomplete Freud adjuvant (21), or AddaVax adjuvant (19).

There were no statistically significant differences between the obtained titers of IgG classes of antibodies recognizing the antigens used for immunization, confirming that the antigen itself did not affect the overall level of production of specific antibodies (**Figure 2D**), however, the lowest titers were detected for SS-MIC1. To investigate this more broadly, we assessed the titers of IgG antibodies recognizing the specific recombinant antigens included in each chimeric antigen. It should be noted that for each antigen we detected higher titers of IgG antibodies recognizing SAG2 than SAG1, significant in the case of SS-MIC1. This suggests that the SAG2 antigen may contain more antigenic B cell epitopes, stimulating a stronger humoral response. These results are in line with epitope prediction analyses with the BepiPred 2.0 random forest algorithm, where the average predicted residue scores of the SAG2 is 0.514 and SAG1 is 0.479 (**Supplementary Figure S3**). SS-MAG1 has the highest titer to the terminal antigen than the rest of the antigens, especially compared to SS-MIC1. The second highest titer to terminal antigen was in the case of SS-P35. If we investigate the BepiPred 2.0 analysis results (**Supplementary Figure S3**) we can notice that MAG1 and P35 have the highest average residue scores, 0.563 and 0.566 respectively, with long uninterrupted epitopes at a threshold of 0.5, compared to the ROP1 or MIC1. The MAG1 antigen itself has been suggested as a good marker for acute toxoplasmosis, reacting more strongly with antibodies at the early phase of the infection (45).

We also assessed the ability of the produced antibodies to inhibit *T. gondii* growth *in vitro*, alone or due to activation of the complement system (**Figure 2C**). Antibodies alone inhibited parasite development *in vitro*, only in the case of SS and SS-MAG1 immunized mouse sera reducing tachyzoite viability significantly compared to the rest of the antigens and control group. It was previously described that only some monoclonal anti-SAG1 antibodies can inhibit *T. gondii* cell invasion (46). Therefore, the explanation for this phenomenon could be the difference in the epitope specificity of the generated polyclonal antibodies. The antigenic composition may impact the folding of recombinant antigen, masking of epitopes, resulting in different antigen presenting and thus may impact on functionality of generated antibodies. In contrast, the antibodies present in the sera of mice immunized with each protein were able to activate complement through the classical pathway, leading to a cytotoxic effect, significantly inhibiting the growth of parasites, in which case we did not observe significant differences between antigens. Specific antibodies take an important part in defence mechanisms, which include opsonization of the parasite, immune-phagocytosis, inhibition of tachyzoite attachment to host cells or activation of the complement system, and lysis of the parasite (47). Other researchers have developed single-chain fragment variable antibodies against the SAG1 protein demonstrating their ability to inhibit parasite invasion both *in vitro* and *in vivo*, and suggesting their usefulness in therapy (48). It has also been shown that polyclonal antibodies against recombinant PRF can inhibit the parasite invasion *in vitro*, but not completely stop it (49). Similar results were obtained after immunization of animals with recombinant rAMA1, rAMA2 proteins, and AMA-RON complexes, yielding up to 50% reduction in parasite invasion, suggesting that the property of the produced antibodies to inhibit parasite invasion may be a likely mechanism of vaccine protection (50). Considering our results as well as the results of other teams, we believe that testing the functionality of the produced antibodies after immunization is an important result to understand the mechanism of action of potential vaccines.

To assess the induction of the cellular response after vaccination, which plays a key role in the effective control of the parasite (47) we firstly determined the T-cell CD3^+^ populations in the splenocytes of immunized animals along with the CD4^+^ and CD8^+^ subpopulations. CD4^+^ lymphocytes are of interest because they represent helper T cells, responsible for mediating and polarizing responses to pathogens, while CD8^+^, cytotoxic lymphocytes, have an important role in parasite clearance. Secondly, we performed stimulation of splenocytes from immunized and control mice *ex vivo*, with native *T. gondii* antigens to assess the response of antigen-specific T cells, and their ability to produce Th1 (IFN-γ, IL-2) and Th2 (IL-10) cytokines.

Immunization of the animals resulted in a statistically significant increase in the lymphocyte population (CD3^+^) but only for the SS, SS-MAG1, and SS-ROP1 groups. The population of T helper cells (CD4^+^) increased statistically significantly for the SS and SS-MAG1 groups while the trend itself is comparable to the results for the total T cell population (CD3^+^), with the SS-MIC1 and SS-P35 groups showing no differences compared to the PBS group. For the CD8^+^ cytotoxic lymphocytes, we noticed an increase in this population in all groups, with the SS, SS-MAG1, and SS-ROP1 groups marked as discovery, by analysis. From the results, we can conclude that vaccination caused an increase in the CD3^+^, CD3^+^ CD8^+^, and CD3^+^ CD4^+^ lymphocyte populations, but mainly noticeable in CD3^+^ CD8^+^ population, which is seen in other vaccine trials (51, 52).

Stimulation of splenocytes with native *T. gondii* antigens *ex vivo*, resulted in significant induction of IFN-γ, for each modification of SS antigen with SS antigen alone showing no discovery in FDR analysis despite slightly higher mean value compared to control group. This cytokine is crucial for the expression of IFN-γ inducible genes such as iNOS or indoleamine 2,3-dioxygenase, activation of macrophages/dendritic cells and the development of memory macrophages via the IFN-γ pathway (53). The effector functions of this cytokine are crucial to prevent the reactivation of brain inflammation caused by *T. gondii*. The production profile of IL-2 parallels that of IFN-γ, as this cytokine is mainly produced by Th1 cells and is responsible for stimulating the proliferation and activation of CD8^+^ cells (54), the main producers of IFN-γ, leading to its high levels. High levels of IL-10 production are associated with high levels of IL-2 as well as IFN-γ production, suggesting that their excessive overproduction, is associated with a strong pro-inflammatory response. This confirms that in addition to being typically a Th2 cytokine, it may have a positive effect in controlling the inflammatory response and prevent pathology (55). In the proliferation assay, we noted that the stimulation index for each modification of the SS recombinant chimeric antigen is higher than that of the control group, significantly for SS-MAG1, SS-MIC1, and SS-P35. These results are consistent with the production profile of pro-inflammatory cytokines, which induce lymphocyte proliferation. SAG1/SAG2 chimeric protein expressed in *E. coli* or *Pichia pastoris* expression systems was tested previously as a potential vaccine, to protect against acute toxoplasmosis. In those studies, only cellular response was assessed, and both tested recombinant SAG1-SAG2 antigens induced high levels of IFN-γ production in splenocytes (56, 57), which is in stark contrast to our results where the SS chimera caused a low increase in the production of this cytokine, with no statistical significance detected. The results could be influenced by many factors, one of which could be the difference in the strain of mice.

The most informative part of the vaccine efficacy evaluation is the induction of experimental toxoplasmosis, in immunized and control mice, and the assessment of invasion inhibition, preferably during chronic phase. Of note, humans are mostly infected by the cyst-forming strains and acute, life-threatening acquired toxoplasmosis occurs mainly in immunocompromised individuals or immunosuppressed transplant recipients as a result of reactivation of tissue cysts present in persons own tissues or transplanted organ. Since the parasite cysts persist in host for life, the evaluation of protection against their formation is of paramount importance. Moreover, chronically infected animals are the main source of human infection through the consumption of undercooked meat containing tissue cysts, thus efficient vaccine must protect host from cyst formation to prevent not only primary but also reactivated toxoplasmosis.

Therefore, in our study, we determined the degree of inhibition of invasion based on the formation of *T. gondii* cysts in the brains of immunized and control mice. Mice immunization with each antigen significantly reduced the number of cysts in the brain compared to the control group. The highest levels of invasion inhibition are noted for SS-MAG1, SS-MIC1, and SS-P35 recombinant chimeric antigens, which are also not statistically different from each other, with SS-MAG1 yielding the highest approx. 75% inhibition. The SS recombinant chimeric antigen showed the lowest percentage of invasion inhibition at approximately 48%. Thus, again each modification of the SS core contributed to a higher degree of inhibition of *T. gondii* invasion. The previously described *in vivo* studies on SAG1-SAG2 recombinant chimeric antigens, produced in *P. pastoris* and *E. coli* systems, efficacy against acute toxoplasmosis gave survival rates of 77 and 73% respectively (56, 57). As shown in our work, the survival studies based on the determination of how many infected individuals survived acute phase of infection do not necessarily reflect the protection against chronic invasion which underscores the importance of chronic invasion experimental model.

The SS-MAG1, SS-MIC1 and SS-P35 antigens ultimately achieved similar protection results, however it is important to underline that regardless of how many immunity parameters are evaluated they represent only a small portion of all reactions triggered by immunization. Based on our results, we propose the SS-MAG1 and SS-MIC1 antigens as the most immunogenic due to their high capacity to inhibit *T. gondii* invasion *in vivo* through potent activation of APC cells, induction of functional antibodies generation, particularly by SS-MAG1, and generation of active memory cells that produce cytokines such as IFN-γ in response to re-exposure.

Interestingly, the results of APC cells stimulation with the tested recombinant chimeric antigens align the most with the obtained results of vaccination efficiency which again points out to the importance of preliminary *in vitro* immunogenicity assessment of potential antigenic vaccine components before moving on to *in vivo* studies.

In conclusion, the addition of another antigenic components to the SS core contributed to its increased antigenicity (11), immunogenicity as well as immunoprotective capacity. Creating an efficient, universal vaccine meant also for humans will require the utilization of already existing data on parasite components immunogenicity to select the most potent and effective antigen combination. Our results, again underline the fact that multi-antigenic preparations usually prove more efficient *in vivo* and chronic infection model is the most suited for selection of antigenic components capable of preventing tissue cyst formation, and thus persistence of the parasite within host cells.

## Data Availability

The original contributions presented in the study are included in the article/**Supplementary Material**. Further inquiries can be directed to the corresponding authors.
