# Supplementary material for: Immunogenicity and protective efficacy of recombinant chimeric antigens based on surface proteins of Toxoplasma gondii
Source: Front Immunol. 2024 Dec 13;15:1480349. doi: 10.3389/fimmu.2024.1480349 (PMC11670819; doi:10.3389/fimmu.2024.1480349)
Supplement: Supplementary file 1 [file DataSheet1.pdf]

Supplementary Material

1 Supplementary Figures and Tables

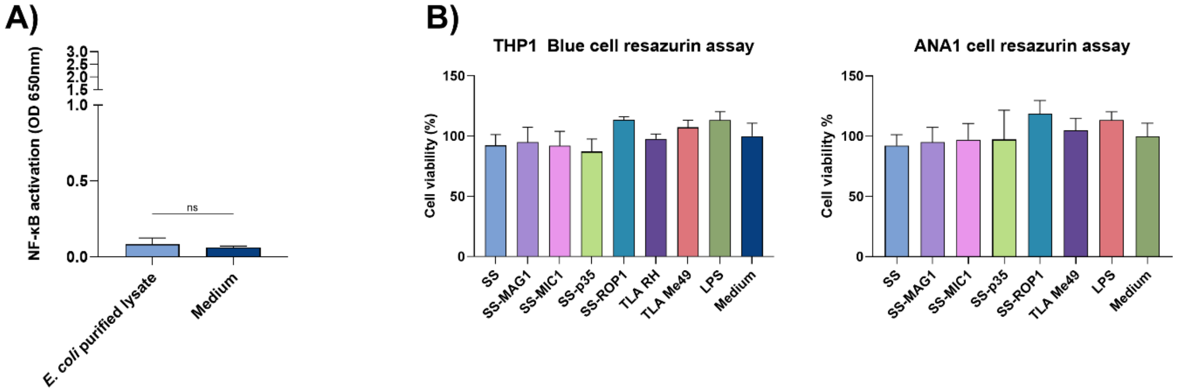

Supplementary figure 1. Supplementary data of the results of *in vitro* stimulation of APC cells. A) Relative activation of the NF-κB pathway (OD 650 nm) after stimulation of THP1-Blue monocytes with *E. coli* Rosetta(DE3)pLysS transformed with empty pET30 Ek/LIC, induced and purified on a metal affinity column, following standard protocol as for antigen purification. Lysate is diluted the same as the least concentrated antigen. Medium - unstimulated cells. Data presented as mean and SD. Analysis was performed using a two-tailed Mann-Whitney test. **B)** Viability of THP1-Blue and ANA1 cells after 24 h culture with recombinant antigens, TLA from RH (TLA RH) and Me49 (TLA Me49) strain of *T. gondii*, LPS, medium (unstimulated cells). The viability percentage was calculated using 550 nm and 600 nm OD readings. Data presented as mean and SD.

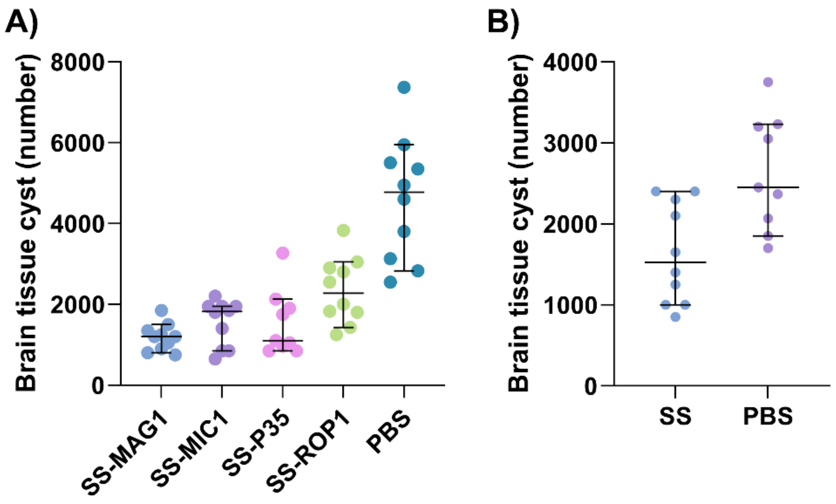

Supplementary figure 2. Brain cyst number per whole brain of vaccinated and infected mice. A) Cyst number per brain of SS-MAG1, SS-MIC1, SS-P35, SS-ROP1vaccinated and PBS (control) mice, after experimental infection with *T. gond*ii DX **B)** Cyst number per brain of SS vaccinated and PBS (control) mice, after experimental infection with *T. gond*ii DX. The SAG1-SAG2 (SS) antigen was performed as a separate experiment and therefore has its own control. These absolute numbers of cysts were used to calculate cyst burden.

MAG1
GeneBank Accession No. XM_002365659.1

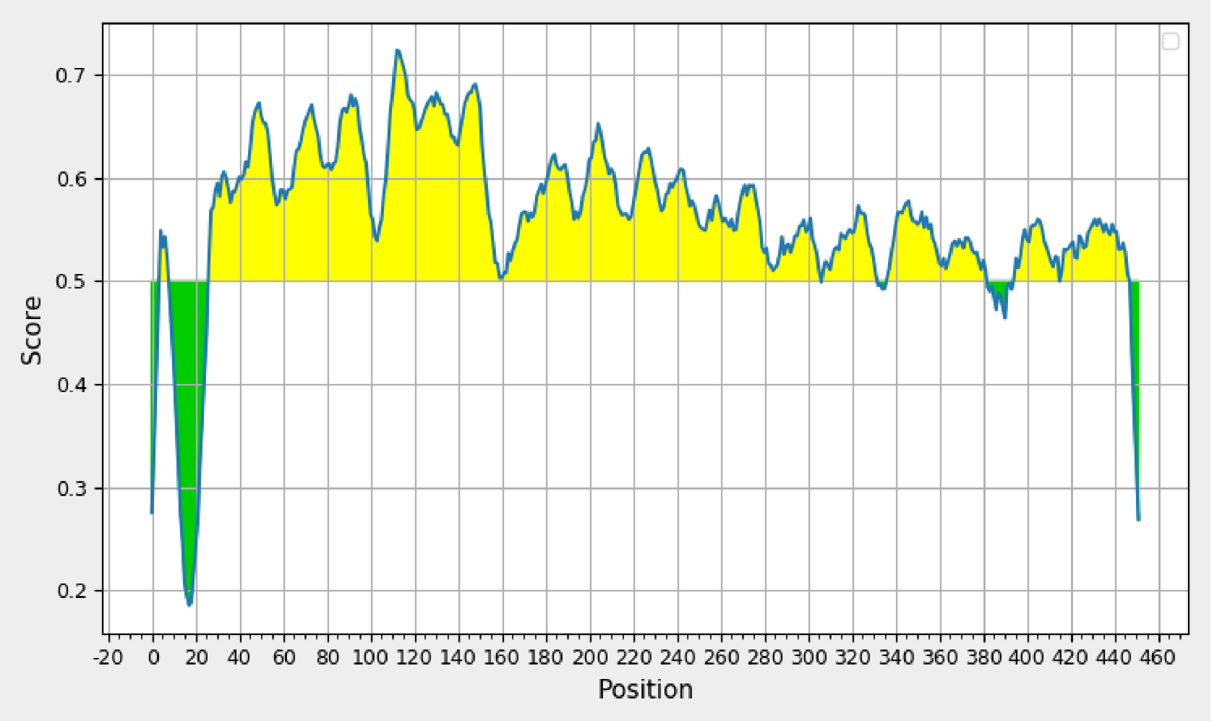

Average: 0.563 **Minimum:** 0.185 **Maximum:** 0.724

No.	Start	End	Peptide	Length
1	5	8	QCRR	4
2	27	306	VGLSQRVPELPEVESFDEVGTGARRSGSIATLLPQDAVLYENSEDVAVPSDSASTPSYFHVESPSASVEAATGAVGEVVPDCEEQQEQGDTTLSDHDFHSGGTEQEGLPETEVAHQHETEEQYGTEGMPPPVLPPAPVVHPRFIAVPGPSVPVPFFSLPDIHPDQVVYILRVQGSGDFDISFEVGRAVKQLEAIKKAYREATGKLEADELESERGPAVSPRRRLVDLIKDNQRRLRAALQKIKIQKKLEEIDDLLQLTRALKAMDARLRACQDMAPIEEA	280
3	308	332	CHKTKAFGEMVSQKAKEIREKAASL	25
4	337	382	GVDAVEKQLRRVEPEHEDNTRVEARVEELQKALEKAASEAKQLVGT	46
5	395	447	TQAVQDSSKDVLTKSQLALVEAFKAIQRALLEAKTKELVEPTSKEAEEARQIL	53

SAG1

GeneBank Accession No. S76248.1

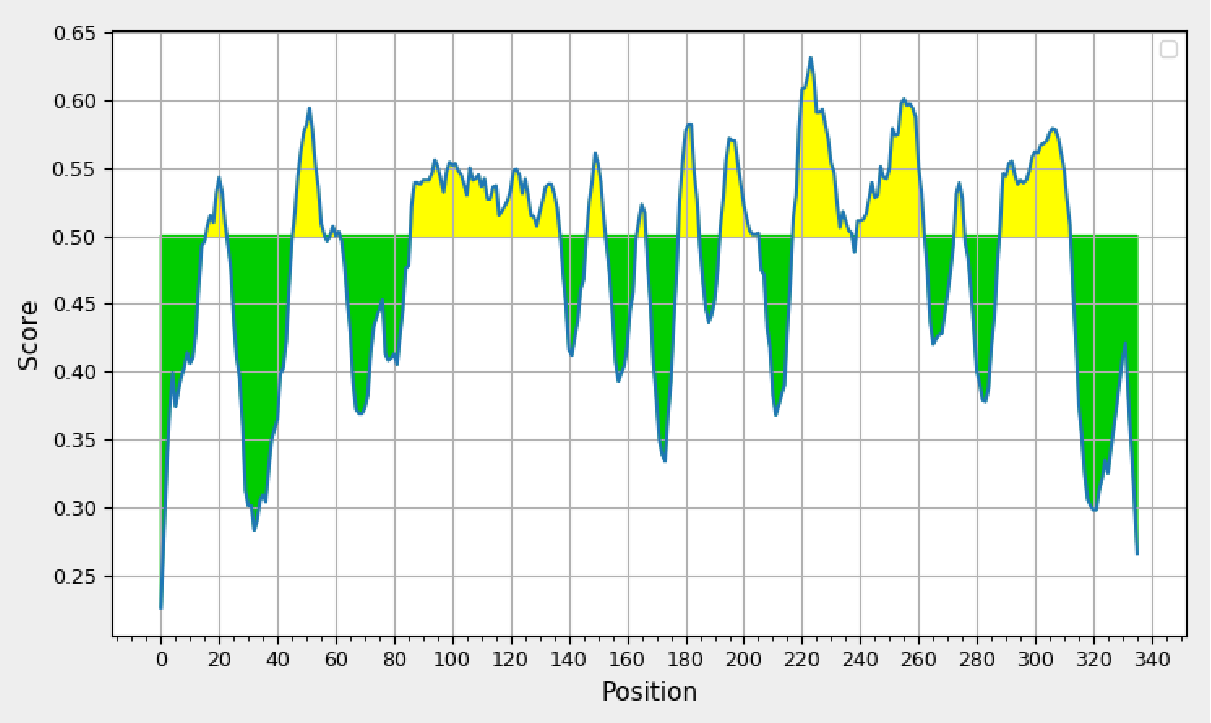

Average: 0.479 **Minimum:** 0.226 **Maximum:** 0.631

No.	Start	End	Peptide	Lenght
1	17	23	SMFPKAV	7
2	47	57	ASDPPLVANQV	11
3	59	62	TCPD	4
4	87	137	LTEPPTLAYSPNRQICPAGTTSSCTSKAVTLSSLIPEAEDSWWTGDSASLD	51
5	148	153	EKFPVT	6
6	165	167	DDA	3
7	179	185	RASSVVN	7

8	193	20<br>6	GADSTLGPVKLSAE	14
9	218	23<br>8	DGVKVPQDNNQYCSGTTLTGC	21
10	240	26<br>3	EKSFKDILPKLTENPWQGNASSDK	24
11	274	27<br>6	FPA	3
12	289	31<br>3	SPEKHHCTVKLEFAGAAGSAKSAAG	25

SAG2

GeneBank Accession No. M33572.1

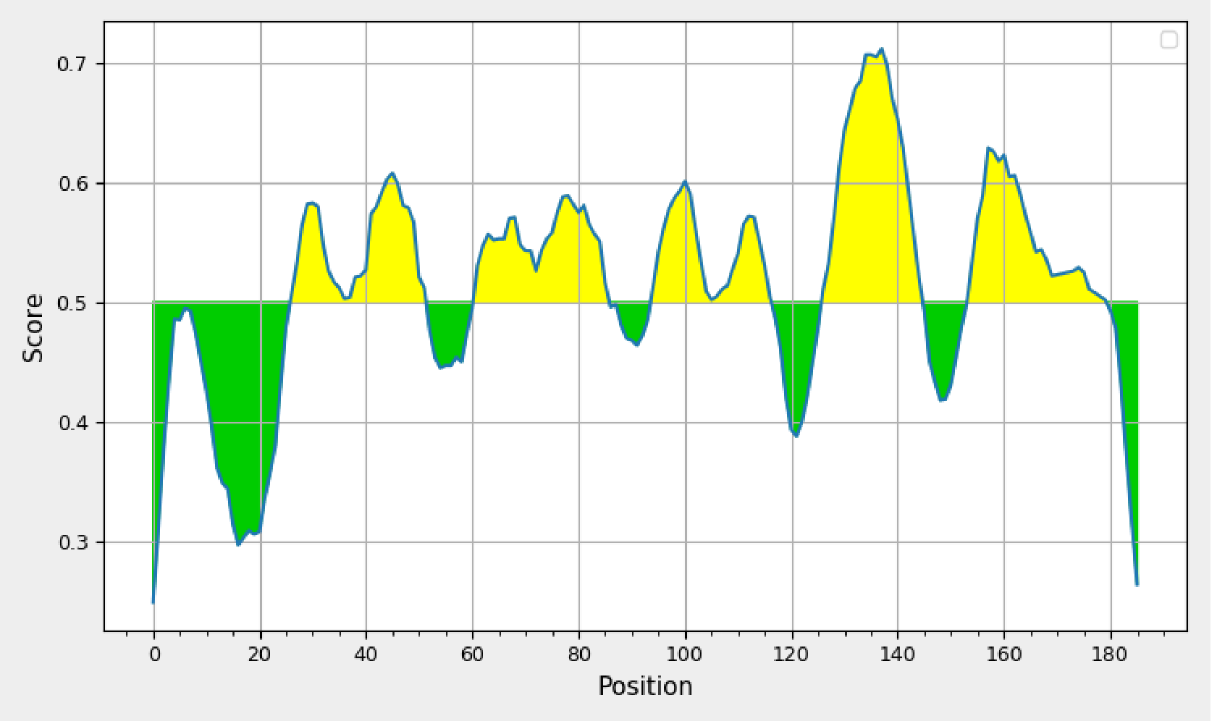

Average: 0.514 **Minimum:** 0.249 **Maximum:** 0.712

No.	Start	End	Peptide	Length
1	27	52	STTETPAPIECTAGATKTVDAPSSGS	26
2	62	86	TISPSGEGDVFYGKECTDSRKLTTV	25
3	95	117	KVQQPAKGPATYTLSYDGTPEKP	23
4	127	145	EAGAPAGRNNDGSSAPTPK	19
5	155	180	GADGRVTSGFDPVSLTGKVLAPGLAG	26

P35

GeneBank Accession No. AF310261.1

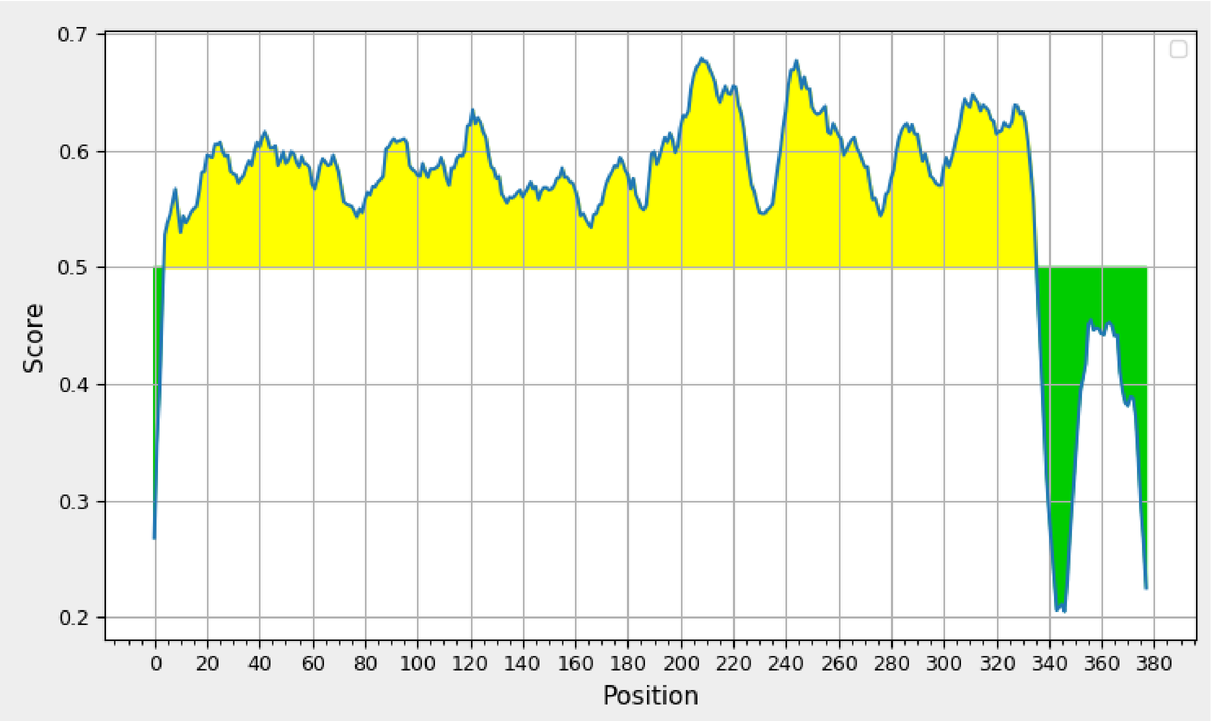

Average: 0.566 **Minimum:** 0.205 **Maximum:** 0.679

No.	Start	End	Peptide	Length
1	5	336	LRVSATVFVVFAVFGVARAMNGPLSYHPSSYGASYPNPSN<br>PLHGMPKPENPVRPPPPGFHPSVIPNPPYPLGTPAGMPQPEV<br>PPLQHPPPTGSPPAAAPQPPYPVGTPVMPQPEIPPVHRPPPPG<br>FRPEVAPVPPYPVGTPTGMPQPEIPAVHHPFPYVTTTTTAAP<br>RVLVYKIPYGGAAPPRAPPVPPRMGPSDISTHVRGAIRRQP<br>GTTTTTTSRKLLFRTAVVAAMAAALITLFRQRPVFMEGVR<br>MFPNLHMPQPEIPAVHHPFPYVTTTTTAAPRVLVYKIPYGG<br>AAPPRAPPVPPRMGPSDISTHVRGAIRRQPGTTTTTTSRKLL<br>F	332

MIC1

GeneBank Accession No. Z71786.1

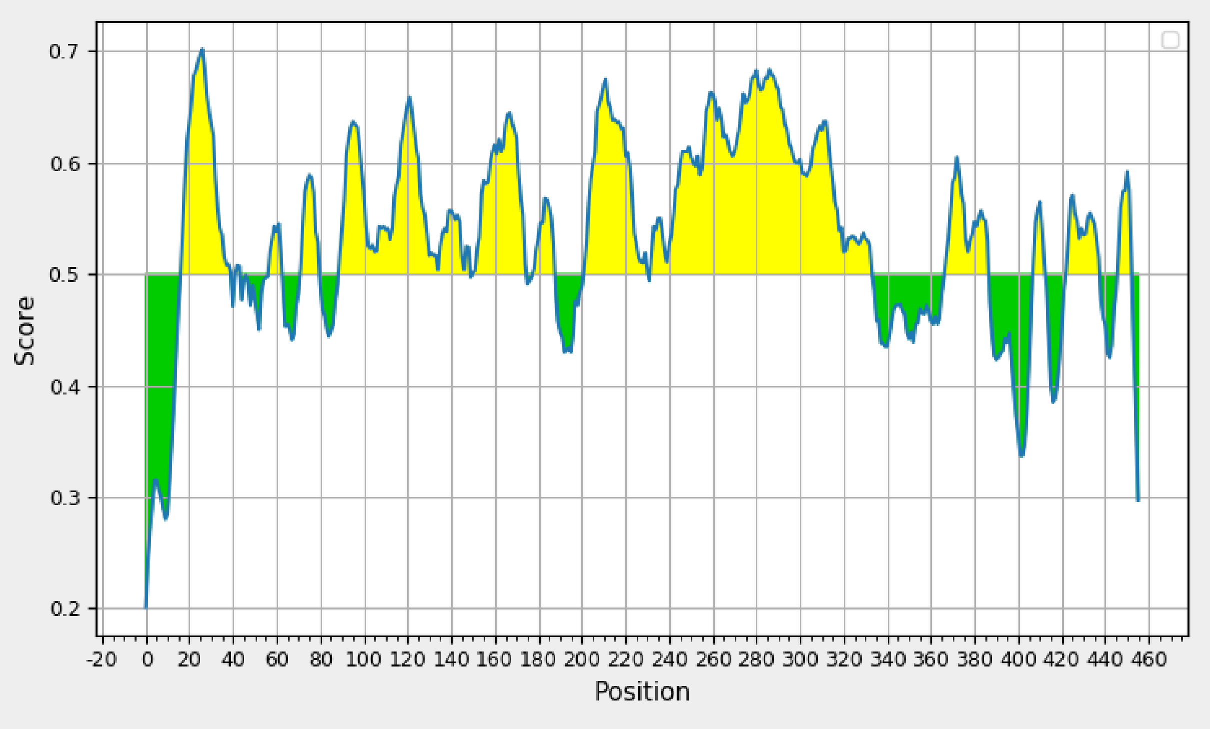

Average: 0.536 **Minimum:** 0.201 **Maximum:** 0.702

No.	Start	End	Peptide	Length
1	17	40	VGPEAYGEASHSHSPASGRYIQQM	24
2	43	44	QR	2
3	58	63	RKMCVP	6
4	72	80	AVGITHQNT	9
5	90	149	SLLESNQENNGVNCVDDCGHTIPCPGGVHRQNSNHATRHEILSKLVEEGVQRFCSPYQAS	60

6	151	175	NKYCNDKFPGTIARRSKGFGNNVEV	25
7	179	188	CYEKASLLYS	10
8	202	231	YCPGGRRGTSTELDKRHYTEEEGIRQAIGS	30
9	233	333	DSPCSEVEVCLPKDENPPLCLDESGQISRTGGGPPSQPPEMQQPADRSDERGGGKEQSPGGEAQPDHPTKGGNIDLPEKSTSPEKTPKTEIHGDSTKATLE	101
10	368	387	FQTGSNSAFDVVEVEEPAGP	20
11	408	413	RLNAAL	6
12	423	438	SGCSSSEEVSFQGVGS	16
13	447	453	LGESPTA	7

ROP1

GeneBank Accession No. M71274.1

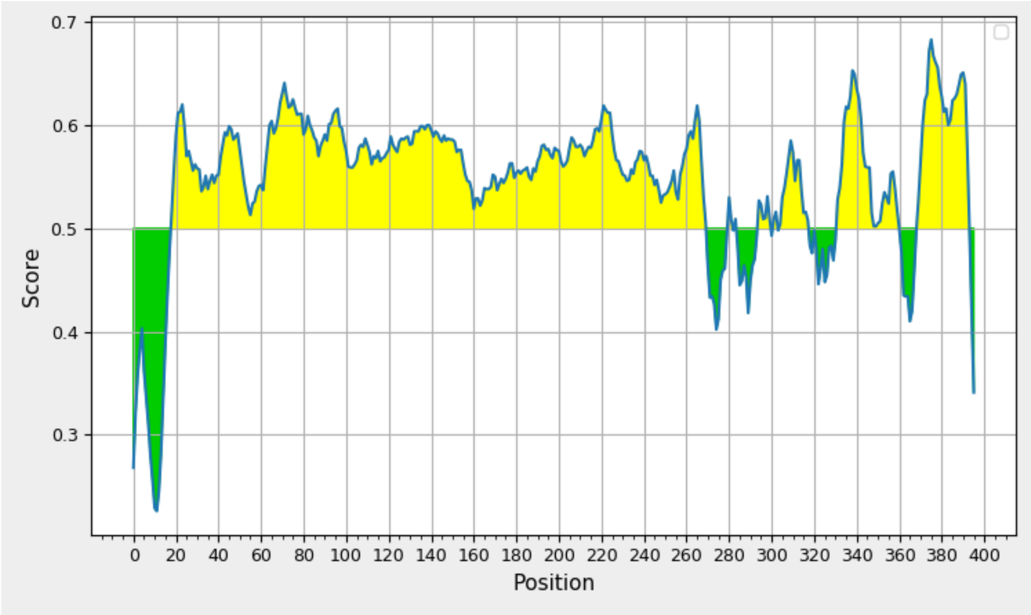

Average: 0.547 **Minimum:** 0.226 **Maximum:** 0.683

No.	Start	End	Peptide	Length
1	19	270	TPSAALSSHNGVPAYPSYAQVSLSSNGEPRHRGIRGTFHMSVKPHANADDFASDDNYEPLPSFVEAPVRGPDQVPARGEAALVTEETPAQQPAVALGSAEGEGTSTTESASENSEDDDTFHDALQELPEDGLEVRPPNAQELPPPNVQELPPPTEQELPPSTEQELPPPVGEGQRLQVPGEHGPQGPPYDDQQLLLEPTEEQQEGPQEPLPPPPPPTRGEQPEGQQPQGPVRQNFFRRALGAARSRFGGARR	252
2	281	282	GG	2
3	284	284	N	1
4	295	300	RRAREG	6
5	302	303	VG	2

6	305	318	VRRLTSGASLGLRR	14
7	332	360	GAVSSGRRRAADGASNVRERFVAAGGRVR	29
8	370	393	LRRRGRTNGEEGRPLLGEGREQDD	24

Supplementary figure 3. BepiPred Linear Epitope Prediction 2.0 analysis of SAG1, SAG2, MAG1, MIC1, P35, ROP1 antigens.

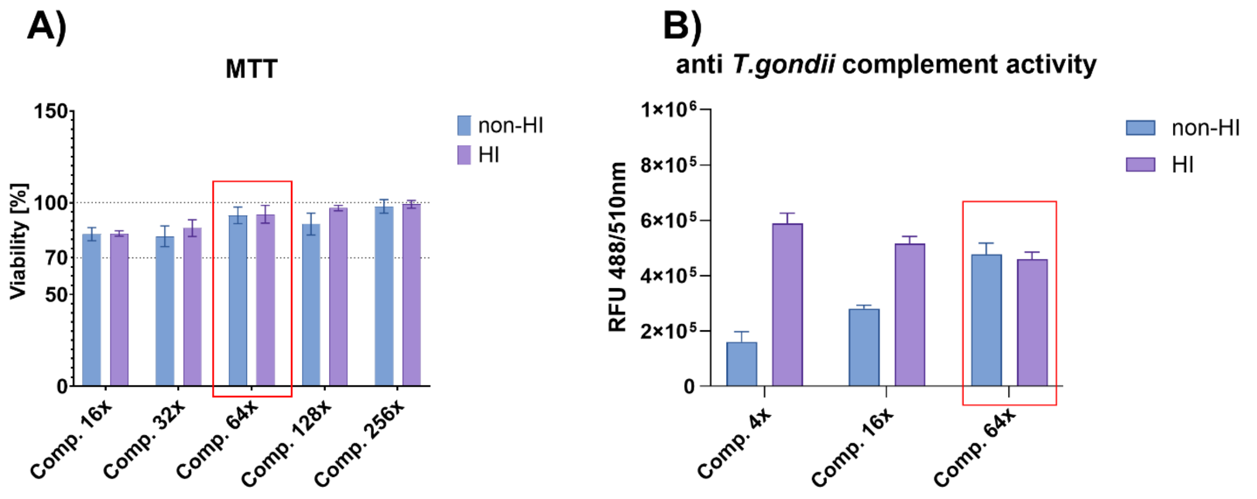

Supplementary figure 4. **Supplementary data of antibody functional assay**. **A)** Viability of Hs27 cells cultured with *Cavia porcellus* heat inactivated and non-heat inactivated serum, at different dilutions, based on MTT assay. **B)** Relative fluorescence intensity of *T. gondii* RH GFP cultured on Hs27 cells for 4 days in the presence of *Cavia porcellus* heat inactivated and non-heat inactivated serum. The red rectangle shows the dilution used in the test chosen based on the anti-*T gondii* activity of the non-inactivated complement. After 64-fold dilution of serum the activity of complement towards tachyzoites was the same as in heat-inactivated serum, which had no effect on *T. gondii* viability. Comp. 4-256x means the dilution factor of complement/*Cavia porcellus* serum. HI- heat inactivated.

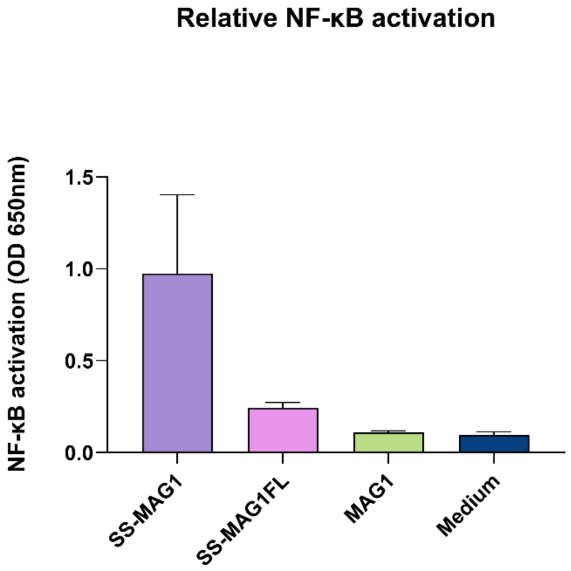

Supplementary figure 5. **Relative activation of the NF-κB pathway** (OD 650 nm) after stimulation of THP1-Blue monocytes with recombinant chimeric antigens, SS-MAG1 (MAG1 30-222 aa), SS-MAG1FL (MAG1 30-452 aa), MAG1 (30-452 aa) or unstimulated cells (medium).

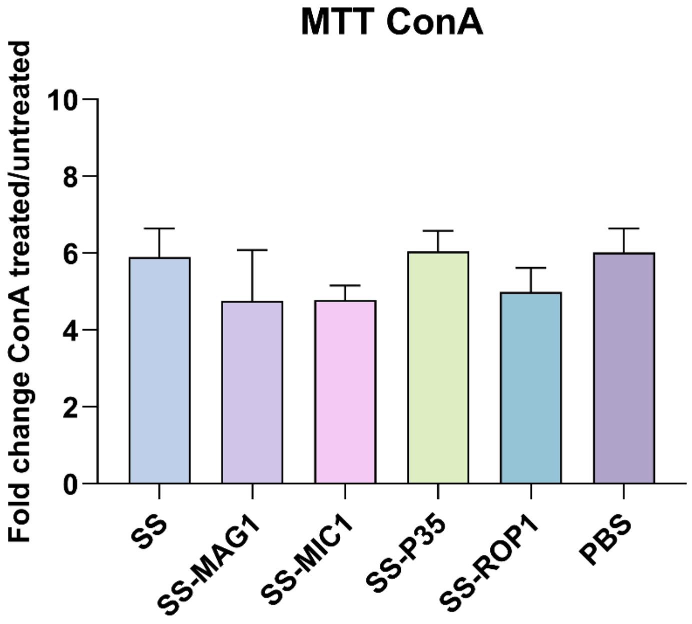

Supplementary figure 6. **MTT test based proliferation response from mouse splenocytes stimulted using Concanavalin A.** Fold change was calculated based on concanavalin treated/ untreated cells mean.

Supplementary table 1. Qualitative analysis of splenocytes ability to produce tested cytokines after Concanavalin A stimulation. In most cases the OD readings for undiluted cell culture supernatants were higher than the OD value for the highest cytokine concentration used to plot standard curve.

cytokine	reactivity of splenocytes from all test groups
IFN-γ	+
IL-2	+
IL-10	+
